# Spermidine Mitigates Immune Cell Senescence and Boosts Vaccine Responses in Healthy Older Adults—A Pilot Study

**DOI:** 10.1111/acel.70545

**Published:** 2026-05-22

**Authors:** Ghada Alsaleh, Mohammad Ali, Amir Hossein Kayvanjoo, Feng Liu, Tanaïs Moreau, Sagida Bibi, Lin Luo, Melissa Govender, Miles Carroll, Sebastian J. Hofer, Eisenberg Tobias, Christoph Magnes, Loren Kell, Christopher Chung, Yu Deng, Aneesha Bhandari, Lucy C. Garner, Thomas Conrad, Liye Chen, Barbara Kronsteiner‐Dobramysl, Susie Dunachie, Owen B. Spiller, Teresa Lambe, Paul Klenerman, Lucy C. Jones, A. Katharina Simon

**Affiliations:** ^1^ Botnar Institute for Musculoskeletal Sciences, Nuffield Department of Orthopaedics, Rheumatology and Musculoskeletal Sciences University of Oxford Oxford UK; ^2^ Kennedy Institute of Rheumatology University of Oxford Oxford UK; ^3^ Peter Medawar Building for Pathogen Research, Nuffield Department of Clinical Medicine University of Oxford Oxford UK; ^4^ Max‐Delbrück Center for Molecular Medicine in the Helmholtz Association Berlin Germany; ^5^ CAMS‐Oxford Institute, Nuffield Department of Medicine University of Oxford Oxford UK; ^6^ Centre for Human Genetics and the Pandemic Sciences Institute, Nuffield Department of Medicine University of Oxford Oxford UK; ^7^ Institute of Molecular Biosciences, NAWI Graz University of Graz Graz Austria; ^8^ Field of Excellence BioHealth University of Graz Graz Austria; ^9^ BioTechMed Graz Graz Austria; ^10^ HEALTH ‐ Institute for Biomedical Research and Technologies Joanneum Research Forschungsgesellschaft Graz Austria; ^11^ Centre for Global Health Research, Nuffield Department of Clinical Medicine University of Oxford Oxford UK; ^12^ Department of Microbiology, University Hospital of Wales, Heath Park Cardiff University Cardiff UK

## Abstract

Older adults are highly vulnerable to infectious diseases, and vaccines are often less effective in this population because of diminished B and T cell memory responses driven by impaired autophagy, immunosenescence, and chronic low‐grade inflammation. Spermidine has been shown to counteract immunosenescence and induce autophagy in preclinical models, and its levels decline with age in humans. We conducted a double‐blind, randomised, placebo‐controlled pilot study in 40 adults over 65 years of age following their third SARS‐CoV‐2 vaccine dose to assess the safety of Spermidine and its effects on vaccine‐induced immunity. Daily oral supplementation (6 mg, 13 weeks) was well‐tolerated. Vaccine non‐responsiveness was common, and non‐responders exhibited a distinct immune‐senescence signature marked by elevated p16, mTOR signalling, and γ‐H2AX+ DNA damage in lymphocytes. Spermidine reversed these features and significantly enhanced spike‐specific IgG secretion, memory B cell recall responses and neutralising antibody activity, specifically in non‐responders. Single‐cell RNA‐seq after treatment revealed increased expression of TFEB targets and autophagy‐related genes in B cells, in line with elevated autophagic flux. These findings suggest that targeting immune cell senescence with Spermidine may improve vaccine responsiveness in older adults and highlight immune‐senescence markers as potential predictors of vaccine failure in ageing populations.

## Graphical abstract

Can we boost vaccine responses in older adults? In a double‐blind, randomised, placebo‐controlled pilot study (*n* = 40, > 65 years), spermidine supplementation (6 mg/day, 13 weeks) was safe and significantly improved immune responses following a 3rd SARS‐CoV‐2 vaccine dose. Vaccine non‐responders showed hallmarks of immune senescence—↑ p16, mTOR signalling, in lymphocytes. Remarkably, spermidine reversed these senescence signatures, restored autophagic flux, and selectively enhanced spike‐specific IgG, memory B‐cell recall, and neutralising antibodies in non‐responders. Takeaway: Targeting immune senescence with spermidine rescues vaccine failure in ageing populations, and senescence markers could help predict poor vaccine responsiveness.

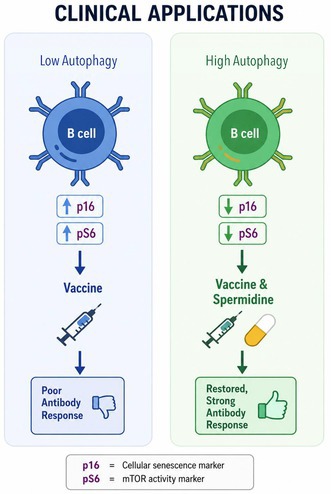

## 1 Introduction

Enhancing immune resilience to existing and emerging infections is key to improving health and extending lifespan in older adults. Impaired immune responses and “inflammaging” (age‐related, chronic, low‐grade inflammation) contribute to age‐related health issues (Franceschi et al. 2000). Collectively, the age‐related gradual dysfunction of the immune system is termed immunosenescence (Teissier et al. 2022), which, along with comorbidities, genetics and environmental factors, weakens vaccine responses. During the SARS‐CoV‐2 pandemic, over 92% of COVID‐related deaths occurred in people aged over 60 (GOV.UK 2023). Vaccination programmes were central to the prevention of infection‐associated morbidity and mortality. However, vaccines often work less effectively in older adults by failing to achieve protective immune responses (Goodwin et al. 2006; Goronzy and Weyand 2013; Osterholm et al. 2012; Palacios‐Pedrero et al. 2022). Instead, they elicit reduced amplitude and duration of immune responses, for example, to hepatitis A and B (Van der Wielen et al. 2006; D'Acremont et al. 2006; Yen et al. 2005), trivalent influenza (Pritz et al. 2015; McElhaney et al. 1998; Bernstein et al. 1998), and tick‐borne encephalitis (Hainz et al. 2005). Similarly, lower neutralising antibody levels against SARS‐CoV‐2 were observed in individuals over 80, regardless of the vaccine platform (Ward et al. 2020; Collier et al. 2021). Furthermore, older adults also have lower T cell vaccine responses (Pritz et al. 2015; McElhaney et al. 1998, 1992; Bernstein et al. 1998; Weinberger 2018). To tackle poor vaccine responses in older adults, targeting immunosenescence may offer a more practical approach than developing novel age‐tailored vaccines for every pathogen (Klaus 2024). Discovering biomarkers for vaccine non‐responders may also improve how we address this group's specific health needs related to vaccines. The key to improving immune resilience in older adults is reducing the “cellular age” of immune cells. This includes addressing issues like replicative senescence, poor mitochondrial health, dysregulated nutrient sensing and poor protein quality control mechanisms (Lopez‐Otin et al. 2023). However, very few studies have examined these hallmarks of ageing (Hofer et al. 2025) in the context of vaccinations.

Autophagy is a promising target for improving immune responses in older people, as it is essential for immune cell function (Clarke and Simon 2019; Metur and Klionsky 2021) and dwindles with age. Autophagy controls protein quality and the regulated breakdown and recycling of damaged cellular components. However, it declines with age, and compromised autophagy has been deemed an indicator of progressive ageing (Schmauck‐Medina et al. 2022) across species (Cuervo and Dice 1998; Sarkis et al. 1988; Hughes and Gottschling 2012; Alsaleh et al. 2020). In line, upregulation of autophagy genes extends organismal lifespan (Pyo et al. 2013; Simonsen et al. 2008; Ulgherait et al. 2014), and the deletion of key autophagy genes in mice leads to premature ageing of the immune system, with diminished numbers or function of haematopoietic stem cells (Mortensen et al. 2011), macrophages (Stranks et al. 2015) and memory CD8^+^ T cells (Xu et al. 2014; Puleston et al. 2014). We previously observed an age‐related decline of autophagic flux in human peripheral CD8^+^ T cells (Alsaleh et al. 2020; Phadwal et al. 2012), which correlates with inferior vaccine responses.

Spermidine is a compound found in foods like wheat germ and has shown promise in improving autophagy in mice (Puleston et al. 2014; Zhang et al. 2019). Research has demonstrated that Spermidine can enhance autophagy in immune cells, particularly in T^28^ and B cells (Zhang et al. 2019). Our studies and others have shown that Spermidine enhances immune cell function by hypusinating eukaryotic translation initiation factor 5A (eIF5A), which in turn facilitates the translation of proteins with challenging motifs, such as polyprolines, and mitochondrial proteins (Zhang et al. 2019; Puleston et al. 2019). In B cells, eIF5A supports the translation of transcription factor EB (TFEB), a key regulator of lysosomal biogenesis and autophagy, and is also necessary for the synthesis of the autophagy‐related protein Atg3 (Zhang et al. 2019; Lubas et al. 2018).

Importantly, we demonstrated that supplementing aged mice with the endogenous metabolite Spermidine improves CD8^+^ T and B cell memory responses (Puleston et al. 2014; Zhang et al. 2019). On the basis of these concepts, we set out to conduct the first clinical study in humans to investigate the safety of Spermidine and its effects on immunosenescence, autophagic function and vaccine responses in older adults.

## 2 Results

### 2.1 A Daily 6 mg Oral Spermidine Supplement Is Tolerated and Safe in Older People

We conducted a randomised controlled trial (RCT) with daily oral spermidine supplementation (6 mg for 13 weeks) following the third booster shot against SARS‐CoV‐2 in older adults. Treatment was initiated approximately after 4 weeks after the booster shot (Table 1) to enhance long‐lasting adaptive memory responses to the vaccine. In the placebo group (*n* = 18), 9 participants were female (50%) and 9 were male (50%), whereas in the Spermidine group (*n* = 20), 13 participants were female (75%) and 7 were male (35%). All participants (*n* = 38) were White British. Day 0/baseline clinical and anthropomorphic characteristics were similar between groups (Table 1). At baseline (Day 0, before treatment with Spermidine) and week 2, no participants in either the placebo or Spermidine groups had a COVID‐19–like illness or tested positive for SARS‐CoV‐2 by RT‐PCR (Table S1). Full blood count and biochemical profiles were comparable between placebo and Spermidine groups at weeks 0 and 2, with no significant differences in haematological parameters (haemoglobin, MCHC, MCV, RBC, HCT, MCH, WCC and platelet count) or biochemical measures (urea and electrolytes, eGFR, liver function tests, creatine kinase, bone profile, lipid profile and iron profile) at either timepoint (Table 2). There were no adverse events or adverse reactions associated with the nutraceutical. Immune profiling by flow cytometry at week 2 also revealed no significant differences between groups, except for a reduction in CD4^+^ central memory cells and an increase in CD8^+^ effector memory populations (Figure S1).

**TABLE 1:** Baseline characteristics of participants.

Parameter	Placebo (*n* = 18)	Spermidine intervention (*n* = 20)
Sex (number and %)		
Female	9 (50%)	13 (65%)
Male	9 (50%)	7 (35%)
Age at vaccination (years)		
Median	71	71.5
Mean	71.8	71.4
Range	66–81	65–77
Ethnicity (number and %)		
White British	18 (100%)	20 (100%)
Body mass index (BMI)		
Median	26	24
Mean	27.27	24.41
Type of vaccine (1st, 2nd, 3rd dose)		
Pfizer/Pfizer/Pfizer	9	18
Astrazeneca/Astrazeneca/Pfizer	5	1
Astrazeneca/Pfizer/Pfizer	2	1
Astrazeneca/Moderna/Pfizer	2	0
History of COVID‐19 illness at day 0 (number and %)	0 (0%)	0 (0%)
History of positive COVID‐19 swab at day 0 (number and %)	0 (0%)	0 (0%)
Confirmed COVID during the study and timepoint (number, % and week)	3 (week 5, 19 and 19)	6 (week 15, 15,11, 24,24)
Concurrent co‐morbidities (number and %)	17 (94%)	18 (90%)
	17 (94%)	18 (90%)
Concurrent medication (number and %)		
Days since 3rd vaccine dose and treatment start (baseline/day 0). Median, (IQR)	61 (47–116)	58.5 (46–82)
Immunosuppression^a^ (number and %)	0 (0%)	0 (0%)

^a^ Includes participants receiving immunosuppressant therapy or illness resulting in immunosuppression.

**TABLE 2:** Changes in biological and haematological parameters at Day 0/baseline and at a follow‐up appointment (week 2) in all participants.

Parameter	Placebo group		Spermidine group		*p*
	Day 0/Baseline	Week 2	Day 0/Baseline	Week 2	
Haemoglobin (g/L)	145.5 ± 14.5	145.4 ± 14.2	140.7 ± 12.8	138.9 ± 11.2	0.745
Haematocrit (L/L)	0.4 ± 0.0	0.4 ± 0.0	0.4 ± 0.0	0.4 ± 0.0	0.407
RBC × 10^12^/L	4.8 ± 0.4	4.7 ± 0.4	4.6 ± 0.4	4.5 ± 0.4	0.335
MCV (fL)	90.3 ± 7.7	91.0 ± 7.3	90.4 ± 3.6	90.7 ± 3.2	0.713
MCH (pg)	30.7 ± 3.3	31.2 ± 3.4	30.4 ± 1.4	30.8 ± 1.6	0.454
MCHC (g/L)	342.3 ± 12.3	339.4 ± 10.0	339.6 ± 9.5	336.7 ± 7.0	0.195
RBDW (fL)	13.0 ± 0.7	12.9 ± 0.8	13.0 ± 0.6	13.0 ± 0.6	0.823
Platelets (x 10^9^/L)	275.7 ± 62.7	266.7 ± 62.2	275.3 ± 81.0	262.7 ± 77.7	0.496
MPV (fL)	10.8 ± 0.8	10.8 ± 0.7	10.7 ± 1.1	10.8 ± 1.2	0.741
WCC (x10^9^/L)	6.7 ± 1.7	6.6 ± 1.9	5.9 ± 1.4	5.6 ± 1.2	0.605
Sodium (mmol/L)	139.0 ± 2.6	139.4 ± 2.8	138.9 ± 2.1	139.5 ± 2.8	0.377
Potassium (mmol/L)	4.5 ± 0.2	4.5 ± 0.3	4.6 ± 0.3	4.6 ± 0.4	0.633
Chloride (mmol/L)	104.1 ± 3.3	103.8 ± 2.7	103.0 ± 2.1	103.5 ± 2.7	0.851
Bicarbonate (mmol/L)	23.1 ± 2.7	24.1 ± 3.0	23.7 ± 2.6	23.8 ± 2.9	0.382
Urea (mmol/L)	5.5 ± 1.5	5.1 ± 1.3	5.9 ± 1.8	5.6 ± 1.2	0.335
Creatinine (μmol/L)	73.2 ± 15.0	72.9 ± 14.0	72.9 ± 15.9	72.0 ± 11.8	0.846
eGFR (ml/min/1.73m^2^)	80.8 ± 11.7	82.9 ± 8.3	79.5 ± 11.0	80.3 ± 9.2	0.541
Bilirubin (μmol/L)	7.8 ± 4.0	8.5 ± 5.1	8.3 ± 4.4	7.9 ± 3.7	0.914
Alk phos (U/L)	89.4 ± 30.2	84.8 ± 24.2	105.4 ± 77.8	96.1 ± 48.8	0.547
ALT (U/L)	25.4 ± 10.1	25.0 ± 5.4	28.8 ± 24.4	26.2 ± 6.7	0.632
AST (IU/L)	25.5 ± 5.7	24.7 ± 5.8	26.2 ± 6.7	0.231	0.231
LDH (IU/l)	203.7 ± 25.1	189.9 ± 23.3	205.2 ± 31.3	192.8 ± 39.1	0.058
CK (U/L)	104.5 ± 62.2	100.4 ± 52.7	101.6 ± 62.4	102.2 ± 52.2	0.895
Gamma GT (U/L)	30.1 ± 17.6	31.6 ± 22.3	64.0 ± 191.2	39.4 ± 87.4	0.631
Total protein (g/L)	70.0 ± 3.0	69.7 ± 3.4	69.9 ± 3.3	68.5 ± 3.4	0.225
Albumin (g/L)	44.5 ± 3.2	44.4 ± 2.7	44.6 ± 2.8	43.1 ± 2.4	0.215
Globulin (g/L)	25.6 ± 3.0	25.3 ± 2.6	25.4 ± 3.4	25.4 ± 2.6	0.857
Calcium (mmol/L)	2.4 ± 0.1	2.4 ± 0.1	2.4 ± 0.1	2.3 ± 0.1	0.133
Corr.calcium (mmol/L)	2.4 ± 0.0	2.3 ± 0.3	2.4 ± 0.1	2.4 ± 0.1	0.191
Phosphate (mmol/L)	1.1 ± 0.1	15.1 ± 61.5	1.1 ± 0.1	1.1 ± 0.2	0.325
Uric acid (μmol/L)	318.5 ± 63.	316.9 ± 59.8	293.1 ± 70.9	287.7 ± 64.7	0.796
RBG (mmol/L)	5.1 ± 1.0	5.1 ± 1.0	5.0 ± 0.8	5.2 ± 1.0	0.672
Triglycerides (mmol/L)	2.0 ± 0.9	1.9 ± 0.7	1.4 ± 0.6	1.5 ± 1.4	0.972
Cholesterol (mmol/L)	5.0 ± 1.3	4.8 ± 0.8	4.8 ± 1.2	4.8 ± 1.3	0.735
Iron (μmol/L)	18.3 ± 6.6	20.6 ± 12.3	14.2 ± 3.3	15.2 ± 5.6	0.395
TIBC (μmol/L)	64.1 ± 6.7	62.8 ± 6.9	58.1 ± 11.2	56.9 ± 8.5	0.496
Transferrin saturation (g/L)	28.9 ± 10.5	32.6 ± 16.9	25.5 ± 7.6	26.8 ± 8.5	0.351

Abbreviations: Alk phos, alkaline phosphatase; ALT, alanine aminotransferase; AST, aspartate transaminase; CK, Creatinine kinase; Corr.calcium, corrected calcium; eGFR, estimated glomerular filtration rate; Fl, femtoliter; g, gram; LDH, lactate dehydrogenase; MCH, mean cell haemoglobin; MCHC, mean corpuscular haemoglobin concentration; MCV, mean cell volume; mmol, millimole; MPV, mean platelet volume; pg, picograms; RBC, red blood cell count; RBDW, red blood cell distribution width; RBG, random blood glucose; TIBC, total iron binding capacity; U/L, units per litre; WCC, white cell count; μmol, micromole.

### 2.2 Spermidine Supplementation Enhances Vaccine Responses in Non‐Responders

**FIGURE 1:**
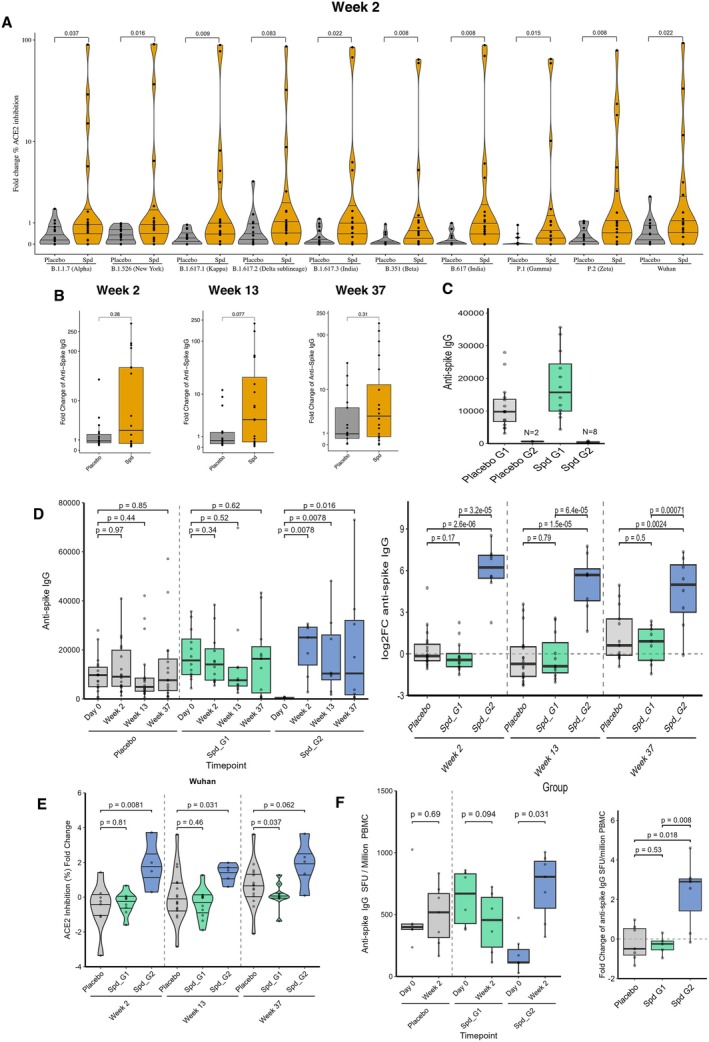
Enhanced vaccine responses observed with Spermidine supplementation. (A) ACE2 inhibition (%) fold change from neutralisation antibody assay in placebo and spermidine groups at 2 weeks, against SARS‐CoV‐2 variants: Wuhan, P.2 (Zeta), B.1.1.7 (Alpha), B.1.617 (India), B.1.351 (Beta), B.1.526 (New York), B.1.617.2 (Delta), B 1.617.1 (Kappa), B.1.617.3 (India), P.1(Gamma). Placebo (grey) and Spd group (orange). (B) Anti‐spike IgG fold change at (left panel) 2 weeks, (middle panel) 13 weeks, and (right panel) 37 weeks post spermidine. Placebo (grey) and Spd group (orange). (C) Anti‐spike IgG intensity (ELISA) at day 0/baseline for the placebo group vaccine responders (R, grey, *n* = 16), placebo vaccine non‐responders (NR, dark grey, *n* = 2), the spermidine‐treated group (Spd) responders (G1, Green, *n* = 12) and Spd non‐responders (G2, Blue, *n* = 8). (D) Anti‐spike IgG intensity (ELISA) at day 0/baseline, 2, 13 and 37 weeks post‐spermidine (Spd) treatment for Spd responders (G1, green), Spd non‐responders (G2, blue) and placebo (grey), raw data (left panel) and Anti‐spike IgG fold change at 2 weeks, 13 weeks, and 37 weeks post‐spermidine compared to baseline (right panel). (E) ACE2 inhibition (%) log_2_ fold change in placebo and both Spd groups across all timepoints compared to Day 0 as measured with the neutralisation antibody assay against the SARS‐CoV‐2 Wuhan variant. (F) Anti‐spike IgG from FluoroSpot for PBMC‐derived B cells at 0 and 2 weeks post spermidine (Spd) for Spd responders (G1, green), Spd non‐responders (G2, blue) and placebo (grey) raw data (left panel) and fold change at 2 weeks post spermidine (right panel). Exact *p* values are reported. Outliers were retained in all statistical analyses; in some plots, extreme values were omitted from display for clarity. Data are presented as Box plots or violin plots showing median, IQR, and 1.5 × IQR whiskers for box plots, with statistical comparisons using the two‐sided Wilcoxon signed‐rank test for B and E and rank‐sum test for A and C. Analyses were conducted in R 4.5.0; α = 0.05.

We first assessed whether serum IgG against the spike protein could inhibit the binding of the spike protein to Angiotensin‐Converting Enzyme 2 (ACE2), the entry receptor for SARS‐CoV‐2, using a pseudo‐neutralising antibody assay. Notably, Spermidine significantly enhanced the blocking antibody response to the spike protein from most viral strains at 2 weeks compared to baseline (Figure 1A). Next, we measured serum anti‐spike IgG levels by ELISA. Although Spermidine treatment increased anti‐spike IgG levels at 2, 13 and even 37 weeks, the effect was not statistically significant (Figure 1B). An important observation from these data showed that only certain participants improved their vaccine response after Spermidine supplementation, whereas others did not. However, analysis of day 0/baseline serum anti‐spike IgG titres, prior to any Spermidine or placebo administration, revealed that 25% of participants were vaccine non‐responders, showing very low or undetectable IgG levels despite having received three doses of a licenced SARS‐CoV‐2 vaccine. Notably, there was an imbalance between the RCT groups: in the Spermidine arm, 8 of 20 participants (40%) were vaccine non‐responders (Group 2, G2). Non‐responders were defined as individuals with anti‐Spike IgG levels below 1000 EU (ELISA Units) at day 0 of the blood draw after the 3rd vaccine dose, whereas the remaining 12 of 20 participants were vaccine responders (Group 1, G1) with detectable IgG titres above that threshold. In contrast, in the placebo group, only 2 of 18 participants were vaccine non‐responders (Figure 1C). This pre‐existing disparity in day 0 vaccine responses between the treatment and placebo groups rendered direct comparisons between the placebo and Spermidine groups challenging. We excluded vaccine type in the Spermidine group as a confounding factor for participants being vaccine responders (G1) or vaccine non‐responders (G2) as most people received three doses of the Pfizer vaccine–only two non‐responders and one responder received a heterologous vaccine schedule. No other obvious parameter (age, sex, ethnicity, history of COVID‐19 illness, co‐morbidity, concurrent medication or time since vaccination) could explain the unresponsiveness to vaccination (Table 3).

**TABLE 3:** G1 and G2 characteristics at day 0/baseline.

Parameter	Spermidine Intervention G1 (*n* = 12)	Spermidine Intervention G2 (*n* = 8)	*p*
Sex (number and %)			
Female	4 (33)	3 (37.5)	0.99^a^
Male	8 (66)	5 (62.5)	0.99^a^
Age at vaccination (years)			
Median	71	72.5	0.99^a^
Mean	71.33	71.37	0.99^a^
Range	65–77	65–77	0.99^a^
Ethnicity (number and %)			
White British	12 (100)	8 (100)	> 0.99^a^
Body mass index (BMI)			
Median	24.5	24	> 0.99^a^
Mean	24.2	24.7	> 0.99^a^
Type of vaccine (1st, 2nd, 3rd dose)			
Pfizer/Pfizer/Pfizer	10	8	> 0.99^a^
Astrazeneca/Pfizer/Pfizer	1	0	> 0.99^a^
Astrazeneca/Moderna/Pfizer	1	0	> 0.99^a^
History of COVID‐19 illness at day 0 (number and %)	0	0	> 0.99^a^
History of positive COVID‐19 swab at day 0 (number and %)	0	0	> 0.99^a^
Confirmed COVID during the study and timepoint (number, % and week)	4 (33%) (week 9, 15, 24, 24)	2 (25%) (week 11 and week 15)	> 0.99^a^
Concurrent co‐morbidities (number and %)	10 (83%)	7 (88%)	> 0.99^a^
Concurrent medication (number and %)	10 (83%)	7 (88%)	> 0.99^a^
Days since 3rd vaccine dose and treatment start (baseline/day 0). Median, (IQR)	68.5 (49–120)	58.5 (31–75)	0.19^b^
Immunosuppression^c^ (number and %)	0 (0%)	0 (0%)	> 0.99^a^

^a^ Categorical variables (vaccine regimen, medications) were compared using Fisher's exact test due to the small sample size.

^b^ Time since vaccine was non‐normally distributed with significant outliers, so median and interquartile range were used with a Mann–Whitney U test.

^c^ Includes participants receiving immunosuppressant therapy or illness resulting in immunosuppression.

We next compared IgG titres in the Spermidine arm on day 0/baseline and then at week 2 after Spermidine supplementation (Figure 1D), stratified for their initial vaccine responsiveness. The 12 vaccine responders had a mean IgG titre of 18,011 (±10,307.33), which did not change significantly after Spermidine supplementation (16,240.5 ± 10,570.70). In contrast, the eight vaccine non‐responders (G2) demonstrated a notable increase in titre from 377.42 (±268.76) to 28,331.02 (±23,976.87) (Figure 1D, left panel), however, these observations should be interpreted with caution given the small sample size. This significant rise in IgG titres persisted at 13 weeks and 37 weeks after the 24‐week washout period (Figure 1D, right panel). The placebo group, including both the responders and non‐responders at baseline, did not exhibit this increase (Figure 1D). Next, for each group, we tested whether serum IgG directed against the spike protein could block the binding of spike protein to Angiotensin Converting Enzyme 2 (ACE2), the entry receptor for SARS‐CoV‐2, using a pseudo‐neutralising antibody assay. Vaccine non‐responders (G2) exhibited a weak inhibition response at baseline (day 0). When analysed as fold changes relative to day 0/baseline, Spermidine treatment induced a pronounced increase in inhibition responses against five of the tested SARS‐CoV‐2 strains, specifically in the vaccine non‐responders (G2), reaching statistical significance at 2 weeks and remaining elevated for certain strains up to 13 and 37 weeks (Figure 1E and Figure S2). This enhancement was not observed in the Spermidine‐treated vaccine responders (G1) or in the placebo group.

To explore the mechanisms underlying the Spermidine‐mediated increase in the vaccine non‐responders, we first analysed B cell responses. We used B cell FluoroSpot assays to enumerate IgG‐secreting cells in memory B cells specific to the spike protein. PBMCs were preactivated with IL‐2 and R484 for 3 days, followed by an ELISpot assay using a plate precoated with spike protein to detect specific antibody‐secreting cells (Figure S3A). As observed previously, on day 0/baseline, vaccine non‐responders (G2) mounted very weak responses compared to the expected stronger day 0/baseline responses of the vaccine responders (G1) and the placebo group (Figure 1F). Strikingly, upon treatment with Spermidine, only the vaccine non‐responders (G2) showed a large increase in IgG‐secreting memory cells at week 2 (Figure 1F). Notably, the total number of memory B cells in blood, identified by CD27 and IgD, was unchanged in flow cytometric analysis (Figure S3B), and Spermidine did not affect total IgA or IgG in this assay (Figure S3C–E), suggesting that it does not have a general impact on the half‐life of immunoglobulins. Together, these results indicate that Spermidine improves the vaccine‐specific antibody‐secreting function of memory B cells in vaccine non‐responders.

Next, given the difference in sex distribution between the spermidine and control groups, we performed sex‐adjusted analyses for all immunological outcomes. The treatment effect remained non‐significant after adjusting for sex (all P_group > 0.23), and sex alone was generally not a significant predictor of outcomes (except for neutralising antibodies at week 37, *p* = 0.042). No significant interactions between sex and the intervention were observed (all P_interaction > 0.45), indicating that the sex imbalance neither confounded nor modified the reported immunological outcomes. A summary of these analyses is provided in Table S2A,B.

We next assessed whether anti–SARS‐CoV‐2 T cell responses were also enhanced in the Spermidine‐treated group. IFN‐γ ELISpot assays on PBMCs, re‐stimulated with S1, S2 or N peptide pools, showed no differences between placebo and Spermidine groups (Figure S3F–H). Within the Spermidine group, no differences in IFN‐γ responses were observed between placebo, vaccine responders (G1) and vaccine non‐responders (G2) at week 2 compared with day 0/baseline (Figure S3F), either in absolute values or fold‐change compared to placebo (Figure S3G,H). No changes were detected at weeks 13 or 37 (Figure S3G). Overall, this shows that Spermidine improves B cell responses in vaccine non‐responders, whereas T cell function remains unchanged.

### 2.3 Spermidine Supplementation Rejuvenates Senescent Cells

**FIGURE 2:**
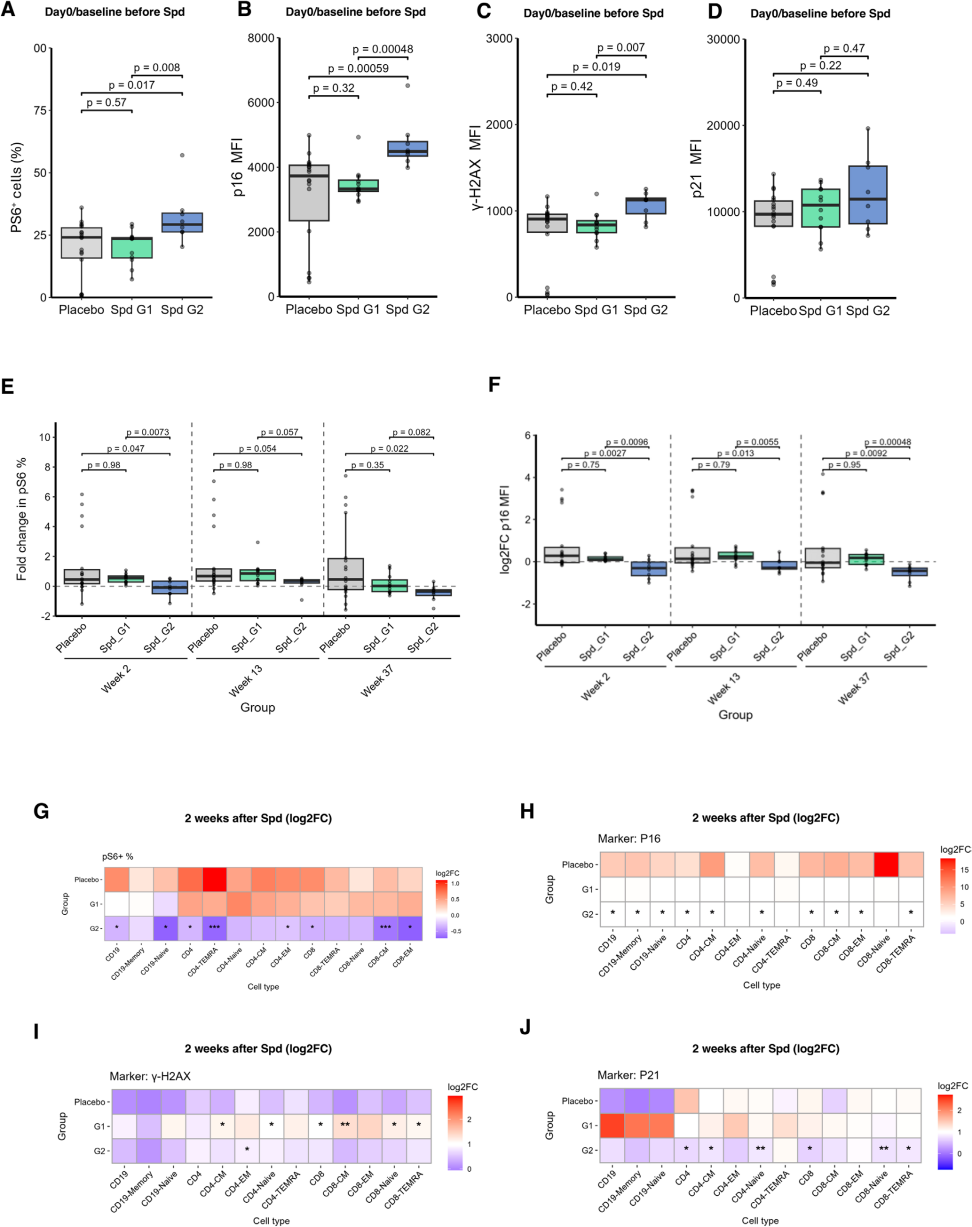
Spermidine treatment promotes senescent cell rejuvenation in vaccine non‐responders (Group 2, ‘G2’). (A–D) PBMCs at Day 0 (baseline). Expression of senescence markers in placebo (grey) and spermidine‐treated (Spd) groups, split into vaccine responders (G1, green) and non‐responders (G2, blue). pS6 is shown as the percentage of positive cells (A), whereas p16 (B), γ‐H2AX (C), and p21 (D) are shown as mean fluorescence intensity (MFI). E–H: Log_2_ fold change between Day 0 (baseline) and Week 2, 13 and 37 for (E) pS6 (% positive cells), (F) p16 (MFI). (G–J) Heatmap showing log_2_ fold change between Week 2 and Day 0 (baseline) for (G) pS6 (% positive cells), (H) p16 (MFI), (I) γ‐H2AX (MFI), and (J) p21 (MFI) in B cell, CD8 T cell, and CD4 T cell subsets. Data are shown as Tukey boxplots (A–F) or heatmaps (G–J). Exact *p* values are reported. Outliers were retained in all statistical analyses; in some plots, extreme values were omitted from display for clarity. For A‐J, statistical comparisons were performed using the two‐sided Wilcoxon rank‐sum test. Statistical significance is indicated by asterisks (*, **, ***, **** for *p* < 0.05, 0.01, 0.001, 0.0001, respectively). Analyses were performed in R 4.5.0 with α = 0.05.

An important observation from the preceding data was that only the vaccine non‐responders improved their vaccine response following Spermidine supplementation, whereas the vaccine responders did not. Interestingly, all eight non‐responders (G2) after the three vaccine doses exhibited elevated levels of a senescence‐associated phenotype prior to supplementation at day 0/baseline (Figure 2A–C). This was reflected by significantly increased phosphorylation of S6 (pS6, a downstream target of mTORC1), along with higher expression of the key cellular senescence markers p16 (*CDKN2A*) and γ‐H2AX (a marker of DNA damage), compared with vaccine responders (G1) and placebo. This suggests that vaccine non‐responders have a unique immune cell senescence signature that could serve as a biomarker to help identify older adults who do not respond well to vaccines. Importantly, Spermidine treatment significantly reduced pS6, p16, and γ‐H2AX levels after 2 weeks compared with baseline/day 0 in vaccine non‐responders, but not in the placebo arm. These reductions were maintained after 13 weeks of Spermidine supplementation and remained evident after the 37‐week washout period (Figures 2E,F and S4A). These findings suggest that Spermidine may increase the vaccine response by decreasing immune cell senescence in whole PBMCs and across different immune cell subsets (Figure 2G–I). In addition, although p21 (*CDKN1A*) levels were not substantially different from responders at day 0/baseline (Figure 2D) they were significantly reduced by Spermidine supplementation compared to placebo (Figures 2J and S4B).

### 2.4 Spermidine Treatment Alters B Cell Pathways

**FIGURE 3:**
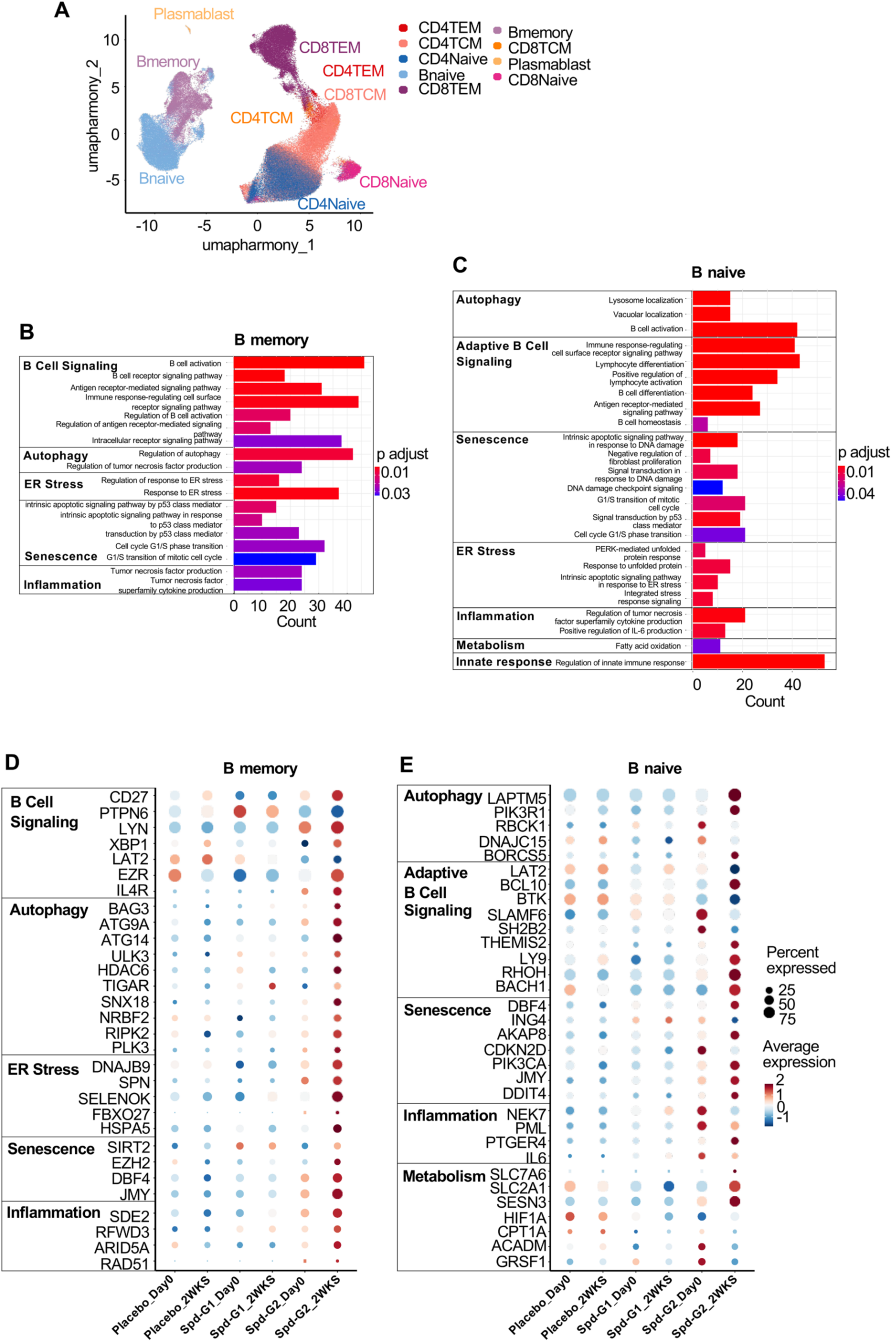
Spermidine treatment leads to significant modifications in B cell pathways and enhances their responses to the COVID‐19 vaccine. (A–C) ScRNA‐seq analysis of 5 volunteers in each group (placebo, Spermidine responders [G1] and non‐responders [G2]) at day 0/baseline (BL) and week 2. (A) UMAP plot of PBMCs, with cell types (colours) identified by multimodal reference mapping using the Seurat annotated reference dataset. (B, C) Pathway enrichment analysis of differentially expressed genes (DEGs) in (B) B memory cells and (C) naïve B cells, showing significantly enriched pathways. (D, E) Gene expression (scaled) dot heatmaps and pathways of the same DEGs in (D) B memory cells, (E) naïve B cells, at Day 0/baseline and Week 2. DEGs were selected with the following criteria: Log2FC > 1; log2FC < 1 and *p*‐value < 0.05.

To further explore the molecular differences between vaccine responders and vaccine non‐responders, single‐cell RNA sequencing (scRNA‐seq) was performed on peripheral blood samples collected at day 0/baseline and 2 weeks post‐Spermidine treatment (week 2) from five participants from each group (placebo, responders and non‐responders). For non‐responders, the five individuals with the greatest improvements in antibody titres by Spermidine were selected, whereas for responders and placebo, participants were matched by vaccine type to the non‐responders to ensure comparability between groups. Our cluster analysis successfully identified all anticipated PBMC subsets by utilising reference mapping from a comprehensive multimodal PBMC reference dataset available through the SeuratData package (Hao et al. 2024) (Figure 3A). To ensure the accuracy of our findings, we manually reviewed the clusters identified by this mapping process (Figure S5A). In line with the outcome of our analyses of lymphocyte function, we observed the most significant changes in B cells. Differential expression (DE) analysis identified a considerable number of differentially expressed genes (DEGs) in both memory and naïve B cells when comparing Week 2 vs. Day 0 in all groups (Figure 3B–E). Gene set enrichment analysis (GSEA) of these DEGs in both cell types highlighted several enriched pathways including B cell activation, autophagy regulation and ER stress pathways (Figure 3B,C). Genes related to inflammation were also significantly upregulated in non‐responders (G2) from Day 0/baseline to Week 2 of Spermidine administration (Figure 3D,E). In addition to these biological processes, we found senescence (cell cycle and DNA repair) and metabolism pathways changed in naïve B cells at week 2 (Figure 3C,E).

Dissecting these pathways, we found that positive regulators of BCR signalling/differentiation/survival were significantly increased only in vaccine non‐responders (G2) between day 0/baseline and 2 weeks of Spermidine treatment. These included *CD27*, *LYN*, *XBP1*, *EZR* and *IL4R* in memory B cells (Figure 3D), and *BCL10, BTK, THEMIS2, Ly9, RHOH* and *BACH1* in naïve B cells (Figure 3E). In line with this, negative regulators of BCR signalling such as *LAT2, PTPN6, SLAMF6* and *SH2B2* were downregulated by Spermidine specifically in vaccine non‐responders (G2) (Figure 3D). Notably, Spermidine supplementation was associated with significantly upregulated autophagy‐related transcripts in non‐responders (G2) only, including *ATG14, ATG9*, as well as *BAG3, ULK3, SNX18, NRBF2* and *RIPK2* (Figure 3D). We also observed a transcriptional pattern consistent with reduced senescence, characterised by upregulation of cell cycle and DNA repair‐associated genes (e.g., *DBF4, AKAP8, PIK3CA, JMY, EZH2* and *SIRT2*) and downregulation of genes that restrict cell cycle progression (e.g., *ING4, CDKN2D* encoding p19) (Figure 3D,E). Although some of these genes are annotated within senescence‐related pathway gene sets, their direction of change is consistent with reduced senescence rather than increased senescence. Interestingly, in non‐responders (G2), memory B cells at day 0/baseline had increased expression of *FCER2* (also known as CD23) and decreased expression of *TNFRSF13B* (also known as TACI) compared to responders (Figure S5B,C). This indicates that memory B cells in non‐responders (G2) may have an altered threshold for activation. Furthermore, *SLC18B1*, a putative polyamine transporter (Hiasa et al. 2014), showed higher expression on day 0/baseline in memory B cells from non‐responders (G2) compared to responders (G1) and placebo (Figure S5D). This suggests that memory B cells in vaccine non‐responders (G2) may have a higher demand for Spermidine as they have exhausted their endogenous Spermidine pool.

Taken together, the scRNA‐seq analysis points toward pro‐proliferative and anti‐senescence effects of Spermidine treatment in B cells in vaccine non‐responders. Other DEG signatures included ER stress, metabolism and inflammation, which are tightly linked to autophagy and known to play a key role in B cells that differentiate into plasma cells (Raza and Clarke 2021; Pengo et al. 2013). This may explain the increase in anti‐spike IgG secretion and improved vaccine response after Spermidine. Notably, we did not find any other altered biological process signature in T cells, which confirms our findings that T cells were little affected by the Spermidine treatment in our trial.

### 2.5 Spermidine Supplementation Enhances Autophagy and eIF5A Hypusination

**FIGURE 4:**
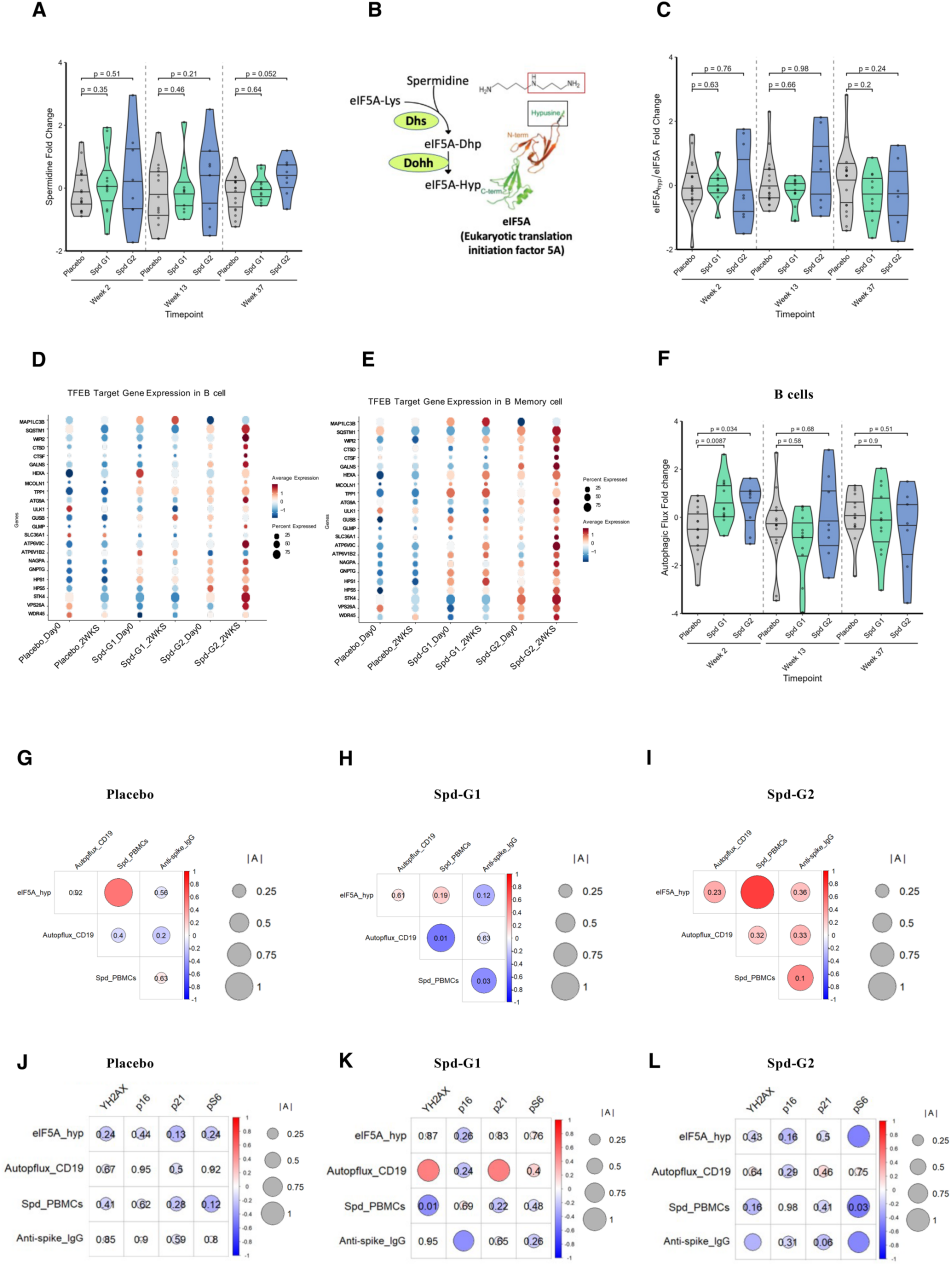
Spermidine supplementation triggers autophagy and correlates with anti‐spike antibody levels. (A) Spermidine log_2_ fold change (Week 2/Day 0) in PBMCs (measured by LC–MS/MS). (B) Schematic representation of the proposed mechanism of action of Spermidine supplementation in eIF5A hypusination. (C) Hypusinated eIF5A log_2_ fold change (Week 2/Day 0) in PBMCs, measured by Western blot. (D, E): TFEB target gene expression (scaled) dot heatmaps and pathways of (D) B cells and (E) memory B cells. Annotations: Spermidine (Spd); responders, group 1 (G1); non‐responders, group 2 (G2). F: Autophagic flux log_2_ fold change at all timepoints post Spermidine supplementation in B cells, placebo (grey), Spd responders (G1, green) and Spd non‐responders (G2, blue). (G–L) Correlation matrices for Placebo (G, J), responders G1 (H, K), and non‐responders G2 (I, L). (A, C, F) Violin plots show median and IQR. Statistical comparisons were performed using the two‐sided Wilcoxon rank‐sum test. Exact *p* values are reported. Outliers were retained in all statistical analyses; in some plots, extreme values were omitted from display for clarity. G, L: Correlation heatmaps show assay–assay associations across weeks 2 and 13. Circle colour represents the LMM‐derived association coefficient (blue = −1, red = 1), whereas circle size corresponds to the absolute value of the coefficient (|A|). *p*‐values are indicated inside circles. Linear mixed‐effects models were used to estimate associations across shared donors for each assay pair; for assay pairs where the LMM could not be fitted (a minority of cases), Spearman correlations were used instead. Analyses in R 4.5.0; α = 0.05.

We previously showed that Spermidine enhances autophagy in immune cells through the eIF5A–TFEB pathway (Gutierrez et al. 2013) (Figure S6A). To understand the mechanism by which Spermidine improves the adaptive immune response to SARS‐CoV‐2 vaccination, we measured Spermidine content in whole PBMCs by mass spectrometry (Figure 4A) and Spermidine is a critical substrate in the hypusination reaction of eIF5A, which is required for its activity as a translation elongation factor, specifically for hard‐to‐translate amino acid motifs (Figure 4B). eIF5A hypusination was assessed by Western blot (Figure 4C). Vaccine non‐responders (G2) exhibited a trend toward higher Spermidine and hypusination levels compared with responders, without reaching statistical significance, compared to responders (G1) and placebo, with an apparent increase at weeks 2 and 13 and potentially a subsequent decrease of hypusination at week 37 after the washout period (Figure 4A,C). Although this did not reach statistical significance, the pattern suggests that with a larger cohort, these changes might become more robust. Next, we examined TFEB targets across lymphocyte subsets in our scRNA data. Upon Spermidine treatment, most TFEB‐regulated genes were significantly upregulated in B cells in vaccine non‐responders after 2 weeks, compared to vaccine responders and placebo (Figure 4D), particularly in memory B cells (Figure 4E), whereas CD8 and CD4 T cells exhibited smaller transcriptional increases (Figure S6B,C), compared to responders and placebo. Taken together, these DEG patterns suggest that Spermidine specifically induces autophagy and the TFEB pathway in B cells of non‐responders (G2) at this timepoint. Notably, among the upregulated genes is *MCOLN1*, which activates calcineurin. Calcineurin dephosphorylates TFEB, leading to its nuclear translocation (Medina et al. 2015). Finally, we used flow cytometry‐based staining for lipidated LC3 to measure autophagic flux, as described previously (Alsaleh et al. 2020). Although no significant differences in autophagy flux were observed between vaccine responders (G1) and vaccine non‐responders(G2) in B cells, CD8^+^ or CD4^+^ T cells at day 0/baseline (Figure 4F; Figure S6D,E), Spermidine supplementation induced autophagy flux specifically in B cells, but not in CD8^+^ or CD4^+^ T cells, compared with the placebo after 2 weeks (Figure 4F; Figure S6D,E). This finding suggests that autophagy may contribute to the enhanced capacity of B cells to produce neutralising antibodies, although a larger sample size is needed to confirm this observation. Notably, this represents the first in vivo demonstration of Spermidine supplementation inducing autophagy in humans.

We next examined correlations between intracellular Spermidine levels, hypusinated eIF5A, anti‐spike IgG titres, and senescence markers across groups. Although little correlation was observed in vaccine responders (G1) and placebo, vaccine non‐responders (G2) showed high positive correlations between autophagy flux, intracellular Spermidine and hypusinated eIF5A (Figure 4G–I), alongside significant negative correlations with senescence markers at week 2 (Figure 4J–L). These findings suggest that Spermidine enhances autophagic flux in lymphocytes, likely via eIF5A hypusination, which may explain the improved vaccine responses.

Collectively, our findings support a model where Spermidine supplementation can enhance vaccine responsiveness in immunosenescent older adults by promoting eIF5A hypusination, especially in those cells with a greater need for extracellular polyamine supply. Our data suggest that Spermidine then enhances autophagy and reduces immune cell senescence. This highlights an exciting avenue for future clinical research at the border of vaccinology and the biology of ageing. Furthermore, senescence markers could serve as predictive indicators of poor vaccine responsiveness in older adults.

## 3 Discussion

This pilot and mechanistic study aimed to evaluate the safety of Spermidine supplementation in older adults, to investigate whether it enhances vaccine responses, and to elucidate the underlying molecular mechanisms. This trial was built on our previous findings that Spermidine restores B and T cell function through autophagy induction in vitro and in preclinical mouse models (Alsaleh et al. 2020; Zhang et al. 2019). Antibody responses, crucial for vaccine efficacy (Pollard and Bijker 2021), depend on functional B cell subsets, which decline with age (Frasca et al. 2016). Our data show that Spermidine treatment is safe and induces autophagy in B cells, without altering B cell subset proportions compared to placebo. Particularly in those volunteers with initially weak responses, Spermidine increased IgG secretion, spike‐specific memory B cells, and enhanced neutralising activity against SARS‐CoV‐2 strains. To our knowledge, this study provides the first preliminary evidence that spermidine supplementation may influence these processes in humans.

In murine B cells, Spermidine acts primarily via the hypusination of eIF5A, which leads to enhanced *TFEB* translation and autophagy (Zhang et al. 2019). Despite our cohort's small size and the possibility of additional involved pathways, our results indicate that the importance of the Spermidine‐eIF5A‐TFEB‐autophagy axis is conserved in the ageing human immune system: upon Spermidine treatment, genes under TFEB control and those mapping to the autophagy machinery were highly enriched across different cell types, with a particularly striking enrichment in B cells. This was associated with an improved vaccine response, highlighting a potentially pivotal role of Spermidine in enhancing immune responses in B cells. Notably, the eIF5a/TFEB pathway is more active in B than T cells and TFEB has higher expression profiles in memory versus naïve cells (Fernandez et al. 2022). In this context, we speculate that the limited dose of 6 mg Spermidine used in our study could be primarily utilised by polyamine‐demanding cell types, such as B cells, to sustain the high synthetic demands of eIF5A hypusination. Future clinical trials should thus investigate whether higher doses would elicit effects in T cells as well.

Although this is one explanation why we see no effect on T cells, either in their immune responses or in their transcriptional profile, another is that perhaps Spermidine affects only the resting memory T cells, which reside in the lymphoid organs and bone marrow that were not sampled here (Hiasa et al. 2014). We also cannot exclude that Spermidine also acts on other cells that we could not characterise, such as Tfh in the germinal center, antigen‐presenting cells, and other non‐immune cells in the microenvironment.

Spermidine's effects were largely observed in a subset of 8 out of 20 participants with low vaccine‐specific IgG levels at baseline (before Spermidine treatment). A key reason may be the elevated senescence markers in seronegatives, as immune cell senescence impacts vaccine efficacy, especially in the elderly (Goronzy and Weyand 2013; Akbar and Henson 2011; Muller et al. 2021; Lord et al. 2024). Notably, 2 weeks of Spermidine reduced senescence markers, including p16 in memory B cells, which is linked to cellular ageing. Spermidine could directly promote autophagic degradation of p16, as previous studies have shown its accumulation when autophagy is inhibited (Coryell et al. 2020). These findings suggest that Spermidine mitigates replicative senescence in aged B cells, potentially enhancing neutralising antibody production and affinity maturation in germinal centers (GCs). Spermidine may either boost GC reactions by increasing proliferation or stimulate memory B cells to enhance antibody secretion. However, it is important not to overlook that spermidine‐associated improvements in senescence and stress markers in specific T cell subsets could enhance helper function and indirectly support B cell differentiation. In this context, transcriptional signatures in B cells may act as earlier or more dynamic indicators of functional changes, which do not necessarily correspond one‐to‐one with classical protein senescence markers measured at the same timepoint.

Our study has several notable limitations. First, our analysis was limited to naïve and memory B cells because of blood sampling constraints, whereas greater effects may be seen in long‐lived plasma cells from bone marrow. Second, because of the urgent distribution and administration of licenced vaccines for the UK vaccination schedule, we were unable to control for the vaccine type administered by immunisation services, resulting in some participants receiving heterologous vaccination, although most had homologous schedules. However, of the people taking Spermidine, only one non‐responder and two responders had a heterologous vaccination history. Third, there was some variation in the interval between the third vaccine dose and the start of Spermidine treatment, as the study was conducted during the height of the pandemic and vaccination timing was outside our control. We consider our data informative for future trials and hypothesis‐generating. We also hypothesise that our observations could be explained by the potential influence of Spermidine on the longevity of the germinal centre responses, which are less dependent on precise vaccination‐to‐treatment timing and not on the initiation of T and B responses. However, we acknowledge that there are important immunological processes that begin immediately after vaccination, which we may have missed addressing with our treatment protocol.

Fourth, the small sample size limits generalizability, and future studies should include larger cohorts with a thoroughly controlled vaccination and treatment timeline. Ideally, with future studies, now that we have reported that some older people have very poor responses to SARS‐CoV‐2 vaccines, participants could be allocated to placebo or Spermidine groups on the basis of their IgG response to the vaccine, so that there are equal numbers of non‐responders and responders in each group and Spermidine. The Spermidine treatment should be commenced at the time of vaccination in all vaccinees, bone marrow or lymph nodes should be sampled, and the trial should test vaccines that are known to be less effective in older adults, such as those against influenza. In addition, as dietary spermidine intake was not controlled or quantified, differences in diet may have contributed to variability in spermidine levels among participants.

Apart from the aforementioned limitations, our study has several strengths, especially the immunological and detailed molecular characterisation of vaccinees at an advanced age combined with an informed intervention to ameliorate signs of immunosenescence. Up until now, low‐dose mTOR inhibition was one of the few interventions demonstrated to enhance immunity in older individuals by attenuating immunosenescence (Mannick and Lamming 2023). In humans, the mTOR inhibitor RAD001 (everolimus) has been shown to improve both B‐ and T‐cell responses to influenza vaccination in older adults (Mannick et al. 2014, 2018). Although a larger Phase 3 trial did not achieve statistical significance in reducing mild respiratory tract infections, there was a clear trend toward improved cellular immune function (Mannick et al. 2021).

Interestingly, rapamycin and Spermidine may act through overlapping yet distinct mechanisms. For example, low‐dose rapamycin treatment in older people decreased p21 expression in immune cells (Kell et al. 2025), which was unaffected by Spermidine, yet elevated in our non‐responders. In contrast, we found a Spermidine‐mediated p16 decrease, suggesting that the two compounds may target different branches of the senescence programme. However, rapamycin has also been reported to reduce p16‐positive dermal cells when administered topically in humans (Chung et al. 2019). Furthermore, the geroprotective effects of rapamycin have been attributed to reducing DNA lesion burden and improving cell survival rather than acting solely through autophagy induction. Similarly, our data raise the possibility that Spermidine's enhancement of vaccine responses might also occur through autophagy‐independent pathways.

Whether Spermidine exerts its effects via direct or indirect mTOR modulation in immune cells remains to be determined. Spermidine led to a decrease in p‐S6, suggesting mTOR inhibition, accompanied by a reduction in the senescence marker p16. We also observed that the Spermidine‐responsive (non‐responder) subgroup showed elevated p‐S6 and senescence markers already at baseline, suggesting that Spermidine selectively restores vaccine responsiveness in immune systems burdened by high senescence and mTOR activity. In line with this, our recent study revealed mTORC1 hyperactivation accompanied by DNA damage and senescence in an ex vivo analysis of aged immune cells (Kell et al. 2025).

Taken together, these findings highlight both commonalities and differences by which Spermidine and rapamycin reverse immune cell senescence. Direct comparative studies, for example, rapamycin versus Spermidine treatment, or combined regimens, together with mechanistic measurements (p‐S6, eIF5A hypusination, autophagy flux) will be crucial to determine whether these interventions act redundantly, synergistically, or through complementary pathways in the ageing human immune system.

Our previous work demonstrated that spermidine regulates autophagy in human immune cells through the eIF5A–TFEB axis (Zhang et al. 2019). In the present study, the increased expression of TFEB target genes and autophagy‐related pathways in B cells following spermidine supplementation is consistent with activation of this mechanism in vivo, extending our earlier mechanistic findings to the context of vaccination in older adults. In conclusion, our study provides initial insights into the potential cellular effects of spermidine in the human immune system. It supports the hypothesis that a daily dose of 6 mg is safe and supports the potential development of Spermidine‐based immune enhancers for older adults. It also advances knowledge on the molecular basis of immunosenescence and how to target it—an emerging field fusing vaccinology and geroscience (Hofer et al. 2025). These data support progression to a later phase trial, with sample size estimation informed by observed effect sizes and refinement of prespecified mechanistic endpoints to more precisely interrogate biological pathways. Additionally, our data promote the idea of utilising immune senescence markers as predictive biomarkers for vaccine responses in older individuals, which could help identify poor responders and address key challenges when vaccinating older adults.

## 4 Materials and Methods

### 4.1 Pilot Study Design

To test the safety and mechanisms of action of Spermidine on immune function in older adults, we conducted a double‐blind, randomised pilot study. Twenty participants over the age of 65 received 6 mg of Spermidine daily, whereas another 20 received a placebo following their third SARS‐CoV‐2 vaccination. We selected a dose of 6 mg/day of spermidine because this level has been reported as safe in previous human studies and falls within the safety limits established by regulatory authorities for adults, including older individuals. The European Food Safety Authority has concluded that supplemental spermidine is safe for adult populations at intakes of up to 6 mg/day. Human studies have investigated spermidine supplementation across a range of doses, from lower amounts delivered via spermidine‐rich extracts (~1 mg/day) to higher doses of purified spermidine (15–40 mg/day), with good tolerability reported. Therefore, the 6 mg/day dose used in this pilot study represents a conservative, safety‐guided starting dose rather than an optimised therapeutic dose (Schwarz et al. 2022; Mackert et al. 2024; Keohane et al. 2024). This mechanistic study also measured immune responses, including spike‐specific IgG levels, memory B cell responses, antibody inhibition of spike‐ACE2 receptor binding, cellular senescence and autophagy levels. This study was designed as a pilot trial primarily to evaluate safety (primary endpoint) and feasibility, and the sample size (*N* = 40) was not powered for formal efficacy analyses. Therefore, the immunological outcomes should be considered exploratory and hypothesis‐generating.

**FIGURE 5:**
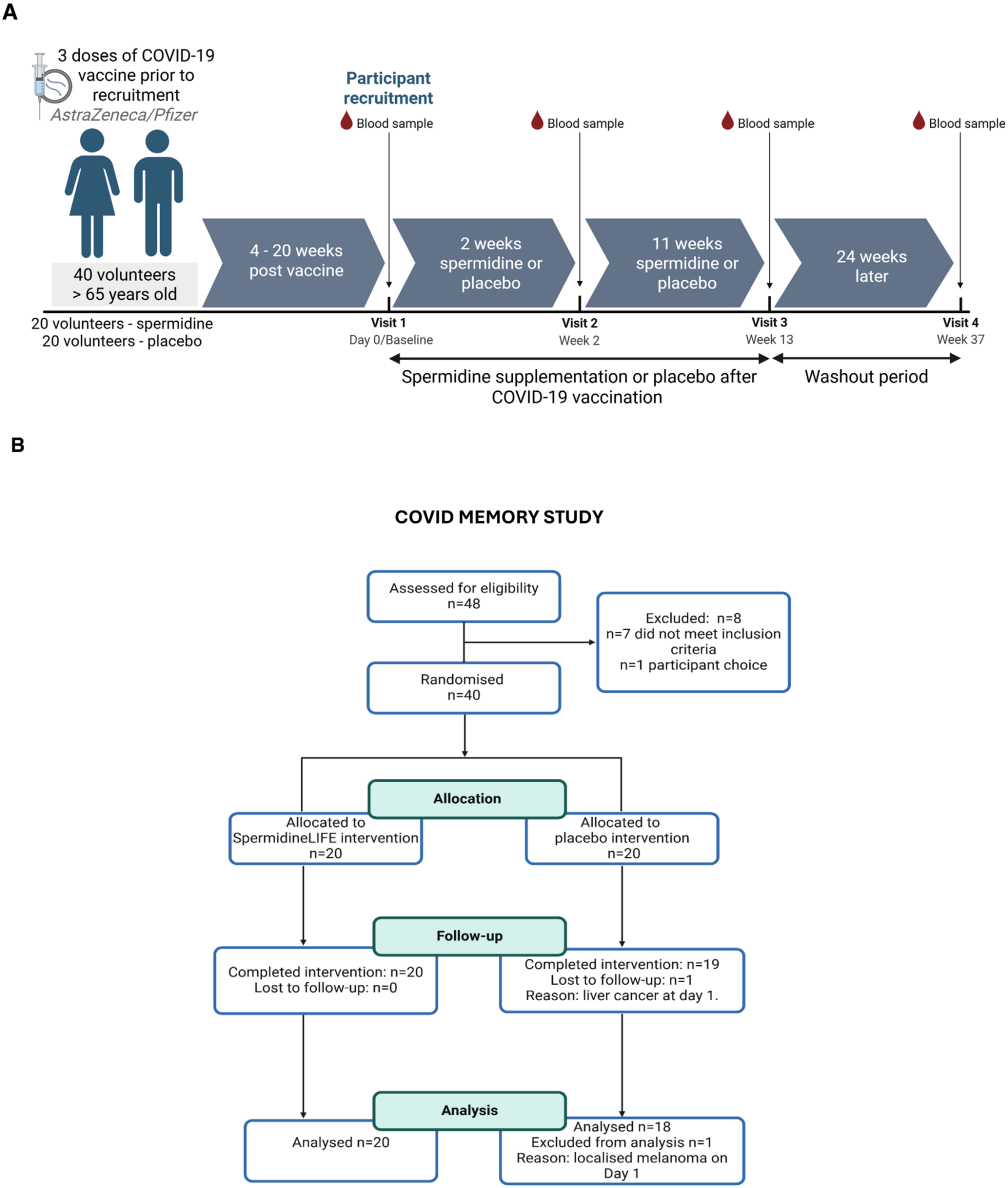
(A) Regimen of immunisation and blood sampling. (B) Study pipeline and participant information.

Our primary endpoint was the safety of a daily oral Spermidine supplement for 13 weeks in older people, after triple SARS‐CoV‐2 vaccination, as well as monitoring its effects on antibody levels in older people. 40 volunteers had already been vaccinated three times prior to this study as part of the UK's national SARS‐CoV‐2 vaccine roll‐out; therefore, the timing of Spermidine treatment had to be adapted at short notice accordingly. The participants were all socially isolating at the time, in accordance with guidance. Because the anti‐spike response peaks in the first week after the third vaccine dose, we proposed that participants join the trial a minimum of 4 weeks before Spermidine or placebo treatment began to avoid effects on innate immunity and the effector phase. Blood samples were taken on day 0/baseline (Visit 1) (before Spermidine administration), and at 2 and 13 weeks after Spermidine or placebo treatment initiation. After a wash‐out period of 24 weeks without any Spermidine or placebo administration, final blood samples were taken at 37 weeks (Figure 5A,B). Our secondary objective was to explore the mechanistic effects of daily Spermidine supplements on the immune ‘memory’ response to SARS‐CoV‐2 vaccine in older people. We examined whether Spermidine improves the immune response to a SARS‐CoV‐2 vaccine and determined the molecular and immunological signature driving immunity, using B and T cell Elispots. We measured autophagy with an autophagic flux assay, measured polyamines in serum and human PBMCs using high‐performance liquid chromatography coupled to mass spectrometry and Single‐cell RNA sequencing was used to measure differential gene expression between groups.

### 4.2 Human Participants and Ethics Statement

40 volunteers aged 65 or older participated in a double‐blind, randomised, controlled, pilot study to determine the safety and effects of daily Spermidine supplementation with Spermidine‐rich wheat germ extract (SpermidineLIFE) on cellular immune responses to SARS‐CoV‐2 vaccination in older adults. The study was reviewed and approved by the University of Oxford, Medical Sciences Research Ethics Committee, under ethical approval R75468/RE003, and was conducted in accordance with the principles of the Declaration of Helsinki, and written informed consent was obtained from all participants prior to enrolment. We registered this study on ClinicalTrials.gov (ID:NCT05421546). This study followed the CONSORT extension guidelines for pilot and feasibility trials. The Spermidine‐rich wheat germ extract (SpermidineLIFE) and placebo were provided by The Longevity Labs GmbH (TLL). Our study examined males and females, and findings are reported for both sexes. Inclusion criteria were age of 65 or more; capacity to provide written consent; and receipt of 3 doses of any licenced COVID vaccine at least 8 weeks before recruitment. Exclusion criteria were: acute illness; use of systemic steroids for more than 1 week, chronic (≥ 14 days in total) administration of immunosuppressants or other immune‐modifying drugs in the 3 months prior to first study intervention; receipt of blood, blood products and/or plasma derivatives or any immunoglobulin preparation in the 3 months prior to first study intervention; immunosuppressive medical conditions or diabetes; allergy to Spermidine supplements, their constituent parts, or gluten intolerance; Spermidine supplementation at the point of recruitment or for 6 months prior. These criteria were determined prospectively. Our primary endpoint was the safety and tolerability of daily Spermidine supplements following vaccination for Coronavirus (SARS‐CoV‐2) and a booster and its effects on antibody levels in older people, using validated assays. Our secondary endpoint was the mechanistic effects of daily Spermidine supplements on the immune ‘memory’ response to the Coronavirus vaccine and a booster in older people. Endpoints and methods were prospectively selected and remained unchanged through the course of the study. The primary objective of this study was to determine if daily Spermidine supplements following vaccination for Coronavirus (SARS‐CoV‐2) and a booster were safe and tolerated, and to measure if Spermidine affects antibody levels in older people. Our secondary objective was to explore the effects of daily Spermidine supplements on the immune ‘memory’ response to the Coronavirus vaccine and a booster in older people. Data generated will be used to perform a power calculation for future larger studies. Objectives were prospectively specified.

Following informed consent, participants were assigned a unique study ID upon recruitment to the trial and randomised in a non‐stratified, double‐blinded manner in a 1:1 ratio to the placebo or Spermidine arm (see Supporting Information). Participants attended 4 research appointments over a 37‐week period at week 0, week 2, week 13 and week 37 (+/− 3 days) (Figure S1).

A clinical case report form recorded details of participants' medical histories. This included: medical conditions; concurrent medication; allergies; COVID infection history and COVID vaccination history (vaccine type, date, number of doses)—all participants received licenced vaccines through the UK COVID‐19 vaccination programme at a time determined by NHS providers; smoking, drug and alcohol use; age; and biological sex. Physical examination was performed at weeks 0 and 2, and abnormal findings were recorded. Anthropometric and vital signs were measured at all visits.

Venous blood (60 mL) was donated at weeks 0 and 2, whereas 50 mL was donated at weeks 13 and 37 with a qualified phlebotomist. BD vacutainers were used to collect venous blood samples for processing of PBMCs (EDTA), serology (SST) and Spermidine (lithium heparin). Samples were processed for PBMC isolation within 3 h of phlebotomy. 10 mL were analysed for urea and electrolytes, liver function tests, eGFR, random blood glucose, creatinine kinase, lipid profile, iron profile and full blood count at weeks 0 and 2, carried out by an accredited UK laboratory. 50 mL were analysed for immune cell activity and immune biomarkers at every timepoint.

Participants were dispensed 13 weeks of Spermidine‐rich wheat germ extract (SpermidineLIFE) or 13 weeks of placebo supplements (rice flour). Participants taking Spermidine‐rich wheat germ extract ingested 6 mg of powdered Spermidine a day for 13 weeks. Participants in the placebo arm ingested the same weight of rice flour. Participants were asked to report any adverse events or reactions to the PI. Compliance with the supplement/placebo, and adverse reactions/events were monitored at the week 2, week and week 13 visits and recorded in a case report form.

Unscheduled visits (remote and in person) were arranged if a participant developed symptoms of COVID or had a positive swab. COVID infection and booster vaccine doses (timing and type of vaccine) were reported at each timepoint for the duration of the trial. Adverse events and reactions were reported to the Sponsor and investigated by the PI at an unscheduled visit as required. Participants' mean age was 72.5 years (median: 71, range: 65–81). 18 participants were male, and 22 were female. All participants were White British. One participant withdrew because of travel difficulties, and a further participant was recruited. Study visits were conducted ±3 days from intended timepoints. At the time of recruitment, week 0 and week 2, all participants reported no previous history of confirmed COVID‐19 or a positive swab or antibody test for COVID‐19. Recruitment to the study began in September 2021 as the third SARS‐CoV‐2 vaccine booster was rolled out across the UK. A sample size of 40 was selected to provide robust feasibility metrics and preliminary biological signals while minimising participant burden in this pilot study.

### 4.3 PBMCs and Serum Preparation

Serum was separated from whole blood from each time point using a 5 mL SSTII BD vacutainer (Becton Dickinson) as per manufacturer's instructions and then stored at −70*°*C in 0.5 mL aliquots until analysed. PBMCs were separated from red blood cells and plasma by overlaying 30 mL of EDTA‐anticoagulated blood (pooled from K2E BD vacutainers) over 15 mL Histopaque‐1077 Hybri‐Max medium in a sterile 50 mL Falcon tube using aseptic methods and then separated by centrifugation at 500 xg, 25°C for 30 min. Following separation, 4 × 1 mL aliquots of plasma were archived at −70°C for later, prior to removing the PBMC layer and washing by centrifugation (800 × g) in sterile, serum‐free RPMI‐1640 culture medium (Sigma‐Aldrich). Cells were counted using an automated Cellometer Vision (Nexcelom Bioscience), quantifying live and dead cells using AO/PI Viastain. PBMCs were then resuspended in sterile foetal calf serum containing 10% DMSO to a final concentration of 10^^6^cells/ml (1 mL aliquots in cryovials) prior to storing in liquid nitrogen until required. One 6 mL lithium BD vacutainer was collected at each timepoint for Spermidine quantification and was separated as above for EDTA samples, except that 0.75 mL plasma was aliquoted, and separation utilised 3 mL Histopaque in a sterile 15 mL falcon tube.

### 4.4 Anti‐Spike IgG ELISA

Serum samples were used to measure and quantify the immunoglobulin G (IgG) antibodies against human SARS‐CoV‐2 spike. Briefly, SARS‐CoV‐2 Spike (Trimer) IgG protein (Native Antigen) was used to coat ELISA plates with 50 μL/well at a concentration of 1 ug/mL overnight. Serum samples were diluted in casein and plated in 96‐well plates in triplicate and left for 2 h in a 20°C incubator. A standard curve was made from a reference pool (1:2 serial dilution) with positive controls. Plates were washed 6 times with DPBS‐T (0.05%). Anti‐human IgG (γ‐chain specific) alkaline phosphatase secondary antibody was added at 1:1000 and incubated for 1 h. Plates were washed 6 times with DPBS‐T (0.05%). 100 μL of pNPP substrate development buffer was added. Plates were read at OD405 nm on the BioTek ELx800 Microplate Reader with Gen5 ELISA software (v3.04).

### 4.5 T Cell Interferon‐Gamma (IFN‐γ) ELISpot Assay

T cell interferon‐gamma (IFN‐γ) ELISpot Assay was performed following the Standard Operating Procedure as published previously (Angyal et al. 2022). After thawing of cryopreserved PBMCs, cells were rested for 3–6 h at 37°C, 5% CO2 in R10 media: RPMI 1640 (Sigma Aldrich, R0883) supplemented with 10% (v/v) Foetal Bovine Serum (FBS, Sigma Aldrich, F7524), 2 mM L‐Glutamine (Sigma Aldrich, G7513) and 1 mM Penicillin/Streptomycin (Sigma Aldrich, P0781). PBMCs were then plated in duplicate at 200,000 cells/well in a MultiScreen‐IP filter plate (Millipore, MAIPS4510) previously coated with capture antibody (clone 1‐D1K, Mabtech, 3420‐3‐1000) and blocked with R10. PBMCs were stimulated with overlapping SARS‐CoV‐2 peptide pools (18‐mers with 10 amino acid overlap, Mimotopes), representing the S1, S2 subunits, membrane (M) and nucleocapsid (N) at a final concentration of 2 ug/ml for 16–18 h in a humidified incubator at 37°C, 5% CO2. CEFT peptide pools (Proimmune, PX‐CEFT) at a final concentration of 2 ug/ml and concanavalin A (ConA, Sigma, 11028–71‐0) at a final concentration of 5 ug/ml were used as positive controls. DMSO (Sigma Aldrich, 67‐68‐5) in R10 was used as the negative control at an equivalent concentration to the DMSO content of the peptide pools. After incubation, cells were removed, and the wells were washed with PBS/0.05% (v/v) Tween20 (Sigma Aldrich, P3563‐5x10PAK). Secreted IFN‐γ was detected by adding 1 μg/mL anti‐IFN‐γ biotinylated mAb (7‐B6‐1‐biotin, Mabtech, 3420–6‐1000) for 2–3 h, followed by 1 μg/mL streptavidin alkaline phosphatase (Vector Labs, SP‐3020) for 1–2 h. Colour development was carried out using the 1‐step NBT/BCIP substrate solution (Thermo Scientific, 34,042) for 5 min at RT. The reaction was stopped by washing the wells with tap water. Air‐dried plates were scanned and analysed with the ImmunoSpot S6 Alfa Analyser (Cellular Technology Limited LLC, Germany). Antigen‐specific responses were quantified by subtracting the mean spots of the negative control wells from the test wells, and the results were expressed as IFN‐γ spot‐forming units (SFU)/million PBMCs. Samples with a high background, defined as a mean spot value greater than 50 in the negative control, were excluded from the analysis. A positive result was defined as greater than 35 SFU/million PBMC, which was calculated by the mean + 2 standard deviations (SD) of the negative control wells included in the analysis across the study.

### 4.6 Memory B Cell FluoroSpot Assay

After thawing, PBMCs were cultured in 96‐well round bottom plates (2 × 10^5^ cells/well) for 72 h at 37°C, 5% CO2, with polyclonal stimulation containing 1 μg/mL R848 and 10 ng/mL IL‐2 from the StimPacl: Memory B cells, Human (Mabtech, 3660–1). Stimulated PBMCs were then added at 2 × 10^5^ cells/well to FluoroSpot plates (Human IgA/IgG FluoroSpotFLEX kit, Mabtech, X‐06G05R‐1) coated with 10 μg/mL SARS‐CoV‐2 spike glycoprotein (The Native Antigen Company, REC31966) and nucleocapsid protein (The Native Antigen Company, REC31851) diluted in PBS (Gibco, 10,010–023), and 1X PBS (negative control wells). In addition, 6.6 × 10^3^ cells/well were added to positive control wells coated with total anti‐immunoglobin at a concentration of 15 μg/mL (Mabtech, FSX‐05R‐1). Plates were incubated for 18 h in a humidified incubator at 37°C, 5% CO2, and developed according to the manufacturer's instructions (Mabtech, X‐06G05R‐1). Analysis was carried out with AID ELISpot software 8.0 (Autoimmun Diagnostika). All samples were tested in duplicate, and memory B cell IgG response was measured as antibody spot‐forming units (SFU) per million PBMCs with the mean spots of the negative control wells (PBS) subtracted.

### 4.7 Neutralisation Antibodies (ACE2 Inhibition Assay)

The percentage of ACE2 inhibition of antibodies against the SARS‐CoV‐2 spike antigens of Wuhan (WT) and other variants (B.1.1.7, B.1.351, B.1.526.1, B.1.617, B.1.617.1, B.1.617.2, B.1.617.3, P.1, and P.2) was determined using the V‐PLEX SARS‐CoV‐2 Panel 13 (ACE2) Kit (Meso Scale Diagnostics, K15466U‐2). The assay was performed according to the manufacturer's instructions, with all incubations at room temperature with shaking at 600 RPM. Briefly, the plates were blocked with Blocker A solution (150 μL/well) for 30 min. Wells were washed three times with 150 μL wash buffer (1×), followed by the addition of samples (25 μL/well, diluted 1:100 in Diluent 100) and the ACE2 Calibration Reagent (25 μL/well) for 1 h. The SULFO‐TAG Human ACE2 Protein was then added to the plate (25 μL/well) for 1 h, after which the wells were washed once more. Finally, MSD GOLD Read Buffer B (150 μL/well) was added, and the plate was read with the MESO SECTOR S 600 instrument.

### 4.8 Surface Staining for Flow Cytometry

PBMCs were plated in a 6‐well plate with 4 mL of R10 and rested overnight. The cells were then washed with PBS containing 5% Foetal Calf Serum (FCS) and resuspended in FACS buffer (PBS; 2% FCS; 5 nM EDTA) comprising various surface marker antibodies (Table S3) at 4°C for 20 min in the dark. Fc block was added to the antibody mix to minimise non‐specific antibody staining. Upon staining, the cells were washed in FACS buffer and either proceeded for intracellular staining or direct acquisition using Cytek Aurora and SpectroFlo software. Gating strategies to determine cellular composition of B cells and CD4^+^ and CD8^+^ T cells (Figure S7A). Acquired data were analysed using FlowJo (v10.8.1).

### 4.9 Autophagic Flux Assay for Flow Cytometry

Autophagy levels in PBMCs were assessed using the FlowCellect Autophagy LC3 antibody‐based assay kit (FCCH100171) and measured after 2 h of treatment with bafilomycin A1 (10 nM BafA1, Cayman Chemical, cat N 11038) or vehicle (DMSO only) (Figure S7B). In brief, cells were stained with surface markers, as indicated above, and washed using Assay buffer in a 96‐well U‐bottom plate. Cells were treated with 0.05% saponin, centrifuged, and then with 1:20 FITC‐conjugated anti‐LC3 antibody in Assay buffer at 4°C for 30 min. The cells were then washed with Assay buffer and fixed with 2% PFA before acquisition using Cytek Aurora and SpectroFlo software. Autophagic flux was analysed using the LC3‐II mean fluorescence intensity of (BafA1‐Vehicle)/Vehicle.

### 4.10 Intracellular Staining for Flow Cytometry

PBMCs were stimulated in R10 using 1 μL of Cell Activation Cocktail with Brefeldin A (Biolegend, Cat N 423304) for 3 h at 37°C. Unstimulated cells were also used as a control. Upon surface marker staining, cells were fixed using 100 μL Fixation buffer (BD, 51‐2090KZ) for 20 min at room temperature in the dark. Following fixation, the cells were washed and permeabilised with 100 μL permeabilization buffer (BD, 51‐2091KZ) for 15 min in the dark and washed again before the intracellular staining. A cocktail of intracellular markers, including senescence markers, was used for staining, as indicated in Table S3, and the cells were incubated in the dark for 30 min at room temperature. The cells were then washed twice with permeabilization buffer and resuspended in FACS buffer before data acquisition. For senescence markers, quantitative analysis was primarily performed using mean fluorescence intensity (MFI), as p16, p21, and γH2AX did not display a clearly distinguishable second peak that would enable reliable gating on the basis of the percentage of positive cells. As illustrated in the gating strategy (Figures S7 and S8), pS6 was the only marker showing a clearly defined second peak. Therefore, the percentage of positive cells was calculated for pS6. Antibody specificity was validated using human dermal fibroblasts treated with 100 nM mitomycin C (MMC) for 6 days to induce cellular senescence. pS6 staining was validated in CD4^+^ cells following treatment with rapamycin.

### 4.11 Detection of Polyamines From Serum and Human PBMCs

Polyamine extraction and quantification using high‐performance liquid chromatography coupled to mass spectrometry (LC–MS/MS) was performed according to^63^ with modifications described in ref 64. Briefly, 100 μL serum was mixed with stable isotope‐labelled standards and subjected to TCA‐based protein precipitation as described (Magnes et al. 2014). For cellular polyamines, one PBMC aliquot was thawed on ice, transferred into a 2 mL protein LoBind reaction tube (Eppendorf) and centrifuged at 1000 rcf, 4°C for 5 min. After centrifugation, the supernatant was removed completely, followed by the addition of 125 μL stable‐isotope labelled standard mix containing 400 ng/mL of each polyamine as reported previously (Magnes et al. 2014). Cells were then resuspended by vortexing and polyamines extracted through the addition of 375 μL 6.25% trichloroacetic acid (TCA) and incubation for 1 h on ice (with vortexing every 15 min).

After TCA extraction, both serum and PBMC samples were centrifuged at 25,000 rcf, 4°C for 10 min, and 150 μL aliquots of the supernatants were transferred to fresh 1.5‐ml LoBind reaction tubes. PBMC cell extracts were processed in duplicates, whereas the remaining supernatant and cell pellet were stored at −80°C for later immunoblotting. Polyamine‐containing supernatants were then derivatised after addition of 37.5 μL ammonium formate (2 M aqueous solution), 800 μL of ultra‐pure water and 125 μL of 1 M Na_2_CO_3_ (aqueous solution adjusted at pH 10 using hydrochloric acid) using 20 μL isobutyl chloroformate (SIGMA) followed by a 15‐min incubation at 35°C. After centrifugation for 1 min at 15,000 rcf, 800 μL supernatant was transferred to LoBind 96‐deep‐well plates (VWR International, 737–2544) and stored at −80°C until further processing.

Preceding LC–MS/MS analysis, polyamine derivatives were extracted by SPE (Strata‐X, Polymeric Reversed Phase, 96‐well plate). SPE was conditioned with 500 mL acetonitrile, equilibrated with 500 mL distilled water containing 0.2% acetic acid. TCA extracts were loaded onto the SPE, and after two washing steps with 500 mL 0.2% acetic acid, samples were eluted with 250 mL 80% acetonitrile containing 0.2% acetic acid. Eluted SPE extracts were subjected to LC–MS/MS (mobile phase: isocratic 80% acetontrile containing 0.2% acetic acid; flow rate 250 mL/min; HPLC column: Kinetex 2.6 mm C18 100A 50 mm 3 2.1 mm and TSQ Quantum Access Max coupled to an Ultimate 3000). MS conditions were set as previously published (Costa‐Machado et al. 2023). LC–MS/MS data were acquired and processed using Xcalibur v.4.0 Software (Thermo Fisher Scientific). Finally, serum Spermidine concentrations were normalised to each patient's day 0/baseline value. For PBMC polyamines, duplicate Spermidine values were averaged (values with > 30% difference between technical replicates (12/154 samples) were excluded), followed by normalisation to each patient's day 0/baseline value.

### 4.12 PBMC Immunoblotting

The pellet after the TCA‐based polyamine extraction was further processed for immunoblotting. After thawing on ice, the remaining polyamine‐containing supernatant was completely removed following centrifugation for 5 min at 10,000 rcf, 4°C. The pellet was then resuspended on ice in 90 μL final sample buffer (62.5 mM Tris/HCl, pH 6.8, 2% sodium dodecylsulfate [SDS], 8.7% glycerol, 0.004% bromophenol blue, 120 mM dithiothreitol), and 10 μL untitrated 1 M Tris solution was added to adjust the pH. The resuspended pellet was vortexed for 10 min at 4°C. After a brief spin in a cooled tabletop centrifuge, the samples were heated for 5 min at 95°C and stored at −20°C upon immunoblotting.

Aliquots used for immunoblotting were thawed on ice and re‐heated just before use. For protein separation, 5 μL were loaded on 4%–12% NuPAGE Bis‐Tris gels (Thermo Fisher Scientific). Electrophoresis was performed at room temperature at 100–140 V with 1× MOPS SDS running buffer (Thermo Fisher Scientific #NP000102). Proteins were wet‐transferred to methanol‐activated 0.45 μm PVDF membranes (Roth # T830.1) at 220 mA for 90 min using transfer buffer (10 mM CAPS/NaOH, pH 11, 10% methanol). After blotting, membranes were blocked with blocking solution (1% dry milk powder in Tris‐buffered saline [TBS] + 0.1% Tween‐20 [TST], pH 7.4) for 1 h and then incubated with the primary antibodies overnight at 4°C. After three washing steps in TST for 5 min, membranes were incubated with secondary, horse radish peroxidase (HRP)‐linked antibodies for 1 h at room temperature. After three washing steps in TST for 5 min, signals were detected with a ChemiDoc detection system (Bio‐Rad) and Clarity Western ECL Substrate (Bio‐Rad) using the ‘optimal exposure’ setting. For re‐probing membranes, Restore PLUS Western Blot Stripping Buffer (Thermo Fisher Scientific #46430) was used according to the manufacturer's protocol. Band intensities were quantified using ImageLab 5.2 (Bio‐Rad) using the rectangular volume tool with local background adjustment. Primary antibodies were anti‐hypusine (Merck #ABS1064‐I, 1:1000), anti‐eIF5A (BD #611977), anti‐GAPDH clone GA1R (Thermo Fisher Scientific #MA5‐15738, 1:10,000). Secondary antibodies were HRP‐linked anti‐mouse IgG (Sigma #A9044, 1:10,000) or HRP‐linked anti‐rabbit IgG (Sigma #A0545, 1:10,000). All antibodies were diluted in TST with 1% dry milk powder.

### 4.13 Single‐Cell RNA Sequencing

#### 4.13.1 Sample Collection and Pooling

Fifteen patient samples (5 per group) for two time points (week 0 and week 2) were collected and divided into two pools for analysis, using 10× Genomics (kit 1,000,414). The single‐cell RNA sequencing (scRNA‐seq) data from these pools were processed and analysed using the Seurat (Hao et al. 2024) and clusterProfiler (Wu et al. 2021) packages in R.

#### 4.13.2 Data Preprocessing and Integration

The scRNA‐seq data from the two pools were initially loaded into R. The datasets from each pool were imported individually. These were then combined into a single Seurat object.

Within the combined object, the RNA assay was processed by joining the layers. The combined dataset was then normalised, and variable features were identified.

Subsequently, the data were scaled, and principal component analysis (PCA) was performed using RunPCA. To correct for batch effects and integrate the datasets, the Harmony integration method was applied to the PCA results through the IntegrateLayers function. Finally, Uniform Manifold Approximation and Projection (UMAP) was run on the integrated data on the basis of the Harmony reduction.

#### 4.13.3 Reference Mapping and Differential Expression Analysis

Seurat v4 Reference Mapping was utilised to annotate the peripheral blood mononuclear cell (PBMC) dataset (Hao et al. 2024, 2021). For differential expression analysis, differentially expressed genes (DEGs) were identified for each cluster. The Seurat object was subsetted for each specific cluster, and the FindMarkers function was used to perform differential expression analysis, comparing conditions at 2 weeks to their respective day 0/baselines. DEGs were considered significant if they had an adjusted *p*‐value (*p*_adj_) < 0.1, using the Benjamini‐Hochberg method.

#### 4.13.4 Functional Enrichment Analysis

To understand the biological implications of the DEGs, functional enrichment analysis was performed using the clusterProfiler package (version 4.6.0) (Wu et al. 2021). For each cluster, significant DEGs were extracted and subjected to Gene Ontology (GO) enrichment analysis using the enrichGO function. The analysis focused on biological processes (BP) with a q‐value cutoff of 0.1, identifying key biological processes associated with the DEGs.

### 4.14 Statistical Analysis

Given this is a pilot mechanistic study and therefore of an exploratory nature, no formal power calculation was performed. Limitations which are inherent in a small sample size, *p*‐values are not intended to provide definitive evidence of statistical significance. Instead, they serve to highlight potential trends or mechanisms that may warrant further investigation in larger, pivotal studies. All statistical analyses were performed in R 4.5.0. For independent group comparisons (e.g., SPD‐G1 vs. SPD‐G2), two‐sided Wilcoxon rank‐sum tests were used. For within‐group comparisons across timepoints (Week 2, Week 13, Week 37 vs. Day 0), two‐sided Wilcoxon signed‐rank tests were applied. Wilcoxon tests used a normal approximation with continuity correction when ties were present. Analyses of log_2_ fold change (log_2_FC) values were performed on the transformed data. Linear mixed‐effects models (lmer, lme4) with Timepoint as a fixed effect and Donor_ID as a random intercept were used for ACE2 inhibition and correlation analyses. *p*‐values for mixed models were calculated via Type III ANOVA (lmerTest). Spearman correlations were applied where mixed models were not possible. The potential influence of sex on all immunological outcomes was assessed by including sex as a covariate in the statistical models, and interactions between sex and the intervention were also tested.

n values indicate biologically independent donors. Data are shown as Tukey boxplots (median, interquartile range, whiskers = 1.5 × IQR), violin plots, or heatmaps. Exact *p* values are reported, with significance threshold α = 0.05. Statistical significance in heatmaps is indicated with asterisks: *p < 0.05, **p < 0.01, ***p < 0.001, ****p < 0.0001.

## Author Contributions

Conceptualization: G.A., L.C.J. and A.K.S. Methodology and investigation: G.A., M.A., S.B., M.G., S.J.H., L.L., E.T., C.M., T.C. O.B.S. and L.C.J. Analysis and visualisation: G.A., A.H.K., F.L., T.M., S.J.H., L.K., C.C., Y.D., A.B., L.C.G., B.K.‐D., S.D., O.B.S., T.L., P.K., L.C.J. and A.K.S. Writing of original draft: G.A., L.C.J. and A.K.S. Editing of the manuscript: all authors.

## Funding

This work was supported by the Arthritis UK grant (22617), Wellcome Investigator award (220784/Z/20/Z), UK SPINE, TLL The Longevity Labs GmbH and Helmholtz Association (REK‐0157) and University of Graz and by the Austrian Science Fund (FWF) grants (P33957, TAI6021000).

## Conflicts of Interest

A.K.S. is a consultant for TLL, The Longevity Labs, GmbH, and Oxford Healthspan. G.A. is a consultant for Oxford Healthspan. T.E. is a consultant for TLL, The Longevity Labs, GmbH. TLL has had no input into the trial design, results, analysis or manuscript.

## Supporting information


**Figure S1:** Immune cell subset profiling between Placebo and Spermidine Groups at 2 weeks (log2FC).
**Figure S2:** Enhanced ACE2 inhibition responses against SARS‐CoV‐2 variants following spermidine supplementation. (A–I) ACE2 inhibition (%) log2FC across all groups and timepoints, from neutralisation antibody assay against SARS‐CoV‐2 variants: (A) B.1.617 (India), (B) P.2 (Zeta), (C) B.1.1.7 (Alpha), (D) B.1.351 (Beta), (E) B.1.526 (New York), (F) B.1.617.2 (Delta), (G) B 1.617.1 (Kappa), (H) B.1.617.3 (India), (I) P.1 (Gamma). Annotations: Week 2 (W2); placebo and spermidine (Spd); group 1 (G1); group 2 (G2). Exact *p* values are reported. Outliers were retained in all statistical analyses; in some plots, extreme values were omitted from display for clarity. Data are presented as violin plots showing median and IQR, with statistical comparisons using the two‐sided Wilcoxon rank‐sum test. Analyses in R 4.5.0; α = 0.05.
**Figure S3:** Spermidine supplementation does not affect memory B cells, antibody levels, or T cell responses following COVID‐19 vaccination. (A) FluoroSpot example image for B cells in spike IgG plates. (B) Memory B cell (CD19^+^ CD27^+^IgD^low^) proportion log_2_ fold change at 2 weeks. (C) FluoroSpot image example results for B cells in IgA and IgG plates. (D) Total IgG (FluoroSpot) log_2_ fold change across groups from baseline at 2 weeks. (E) Total IgA log_2_ fold change across groups from baseline at 2 weeks. (F) ELISpot image example results for T cell plates stimulated with SARS‐CoV‐2 peptide pools representing the viral S1, S2, membrane (M) and nucleocapsid (N) regions. CEFT peptide pools and concanavalin A (ConA) were used as positive controls. (G) Ex vivo IFN‐γ ELISpot response raw data for all groups and timepoints to the total spike. (H) Ex vivo IFN‐γ ELISpot response log_2_ fold change at 2 weeks to total spike. Exact *p* values are reported. Outliers were retained in all statistical analyses; in some plots, extreme values were omitted from display for clarity. Data are presented as Box plots showing median, IQR, and 1.5 × IQR whiskers, with statistical comparisons using the two‐sided Wilcoxon rank‐sum test, except for panel F, where the Wilcoxon signed‐rank test was used. Analyses were conducted in R 4.5.0; α = 0.05.
**Figure S4:** Spermidine treatment promotes senescent cell rejuvenation in vaccine non‐responders, (Group 2, ‘G2’). (A–D) Senescence marker log_2_ fold change between day 0/basline and 2, 13 and 37 weeks for (A) γH2AX (MFI), (B) p‐21 (MFI), in placebo (grey), Spd groups responders (G1, green) and non‐responders (G2, blue) in PBMCs. For A‐B, statistical comparisons were performed using the two‐sided Wilcoxon rank‐sum test. Statistical significance is indicated by asterisks (*, **, ***, **** for *p* < 0.05, 0.01, 0.001, 0.0001, respectively). Analyses were performed in R 4.5.0 with α = 0.05.
**Figure S5:** scRNAseq analysis revealed that spermidine treatment induces significant modifications in B cell pathways. (A) Violin plots showing normalised expression of the top three differentially expressed marker genes per identified cell type. (B–D) Violin plots representing normalised gene expression for select genes differentially expressed between Placebo, G1 and G2 at baseline in the B memory cluster (B) FCER2 (C) TNFRSF13B (D) SLC18B1. Data are presented as violin, dotplots and boxplots with statistical comparisons using the Wilcoxon rank‐sum tests.
**Figure S6:** Spermidine supplementation does not induce TFEB or Autophagy in T Cells following COVID‐19 vaccination. (A) Schematic representation of the proposed mechanism of action of spermidine supplementation in autophagy activation of immune cells. (B, C) TFEB target gene expression (scaled) dot heatmaps and pathways of DEGs in (B) CD8^+^ T cells (C) CD4^+^ T cells. Annotations: Week 2 (W2); placebo and spermidine (Spd); group 1 (G1); group 2 (G2). (D, E) Autophagic flux log_2_ fold change at all timepoints post spermidine supplementation in (D) CD8+ T cells and (E) CD4^+^ T cells in spermidine Placebo (grey) (Spd) groups 1 (G1, green) and Spd group 2 (G2, blue). Annotations: spermidine (Spd); group 1 (G1); group 2 (G2). Exact *p* values are reported. Outliers were retained in all statistical analyses; in some plots, extreme values were omitted from display for clarity. Data are presented as violin plots showing median and IQR, with statistical comparisons using the two‐sided Wilcoxon rank‐sum test. Analyses in R 4.5.0; α = 0.05.
**Figure S7:** Gating strategies to determine cellular composition of B cells and CD4^+^ and CD8^+^ T cells. (A) Gating strategy for CD3^+^, CD19^+^, CD4^+^ and CD8^+^ subsets within live cells. (B) Representative flow cytometry‐based assay for LC3‐II from PBMCs treated with or without bafilomycin A_1_ (BafA_1_) for 2 h prior to staining.
**Figure S8:** Flow cytometry analysis of senescence markers at baseline/day 0. (A–D): Representative flow cytometry for PBMC stained with antibodies targeting senescence markers, (A) pS6 (B), p16, (C) γ‐H2AX and (D) p21 in placebo (grey), and the spermidine‐treated (Spd) groups split into vaccine responders (G1, green) and non‐responders (G2, blue). (E) mTORC1 activity measured by phospho‐S6 (p‐S6) levels was assessed in human CD4^+^ T cells activated for 3 days (1 μg/ml anti‐CD3/28) in the presence of vehicle or 10 nM rapamycin (mTORC1 inhibitor). (F–H) Human dermal fibroblasts were treated with 100 nM mitomycin C (MMC) for 6 days and allowed to recover for a further 6 days to induce cellular senescence. Cells were then stained with antibodies targeting senescence markers, (F) p16, (G) p21 and (H) γ‐H2AX, and measured by flow cytometry.
**Table S1:** Adverse reactions and COVID‐19 cases.
**Table S2A:** Adjusted model (outcome ~ Group + Sex). Tests whether sex imbalance confounds the treatment effect.
**Table S2B:** Interaction model (outcome ~ Group × Sex). Tests whether the treatment effect is modified by sex.
**Table S3:** List of antibodies and reagents.

## Data Availability

The data that support the findings of this study are available on request from the corresponding author. The data are not publicly available due to privacy or ethical restrictions.
