# Supplementary material for: Spermidine Mitigates Immune Cell Senescence and Boosts Vaccine Responses in Healthy Older Adults—A Pilot Study
Source: Aging Cell. 2026 May 22;25(6):e70545. doi: 10.1111/acel.70545 (PMC13240347; doi:10.1111/acel.70545)
Supplement: Supplementary file 1 — Figure S1: Immune cell subset profiling between Placebo and Spermidine Groups at 2 weeks (log2FC). Figure S2: Enhanced ACE2 inhibition responses against SARS‐CoV‐2 variants following spermidine supplementation. (A–I) ACE2 inhibition (%) log2FC across all groups and timepoints, from neutralisation antibody assay against SARS‐CoV‐2 variants: (A) B.1.617 (India), (B) P.2 (Zeta), (C) B.1.1.7 (Alpha), (D) B.1.351 (Beta), (E) B.1.526 (New York), (F) B.1.617.2 (Delta), (G) B 1.617.1 (Kappa), (H) B.1.617.3 (India), (I) P.1 (Gamma). Annotations: Week 2 (W2); placebo and spermidine (Spd); group 1 (G1); group 2 (G2). Exact p values are reported. Outliers were retained in all statistical analyses; in some plots, extreme values were omitted from display for clarity. Data are presented as violin plots showing median and IQR, with statistical comparisons using the two‐sided Wilcoxon rank‐sum test. Analyses in R 4.5.0; α = 0.05. Figure S3: Spermidine supplementation does not affect memory B cells, antibody levels, or T cell responses following COVID‐19 vaccination. (A) FluoroSpot example image for B cells in spike IgG plates. (B) Memory B cell (CD19+ CD27+IgDlow) proportion log2 fold change at 2 weeks. (C) FluoroSpot image example results for B cells in IgA and IgG plates. (D) Total IgG (FluoroSpot) log2 fold change across groups from baseline at 2 weeks. (E) Total IgA log2 fold change across groups from baseline at 2 weeks. (F) ELISpot image example results for T cell plates stimulated with SARS‐CoV‐2 peptide pools representing the viral S1, S2, membrane (M) and nucleocapsid (N) regions. CEFT peptide pools and concanavalin A (ConA) were used as positive controls. (G) Ex vivo IFN‐γ ELISpot response raw data for all groups and timepoints to the total spike. (H) Ex vivo IFN‐γ ELISpot response log2 fold change at 2 weeks to total spike. Exact p values are reported. Outliers were retained in all statistical analyses; in some plots, extreme values were omitted from display for clarity. [file ACEL-25-e70545-s001.pdf]

Figure S1

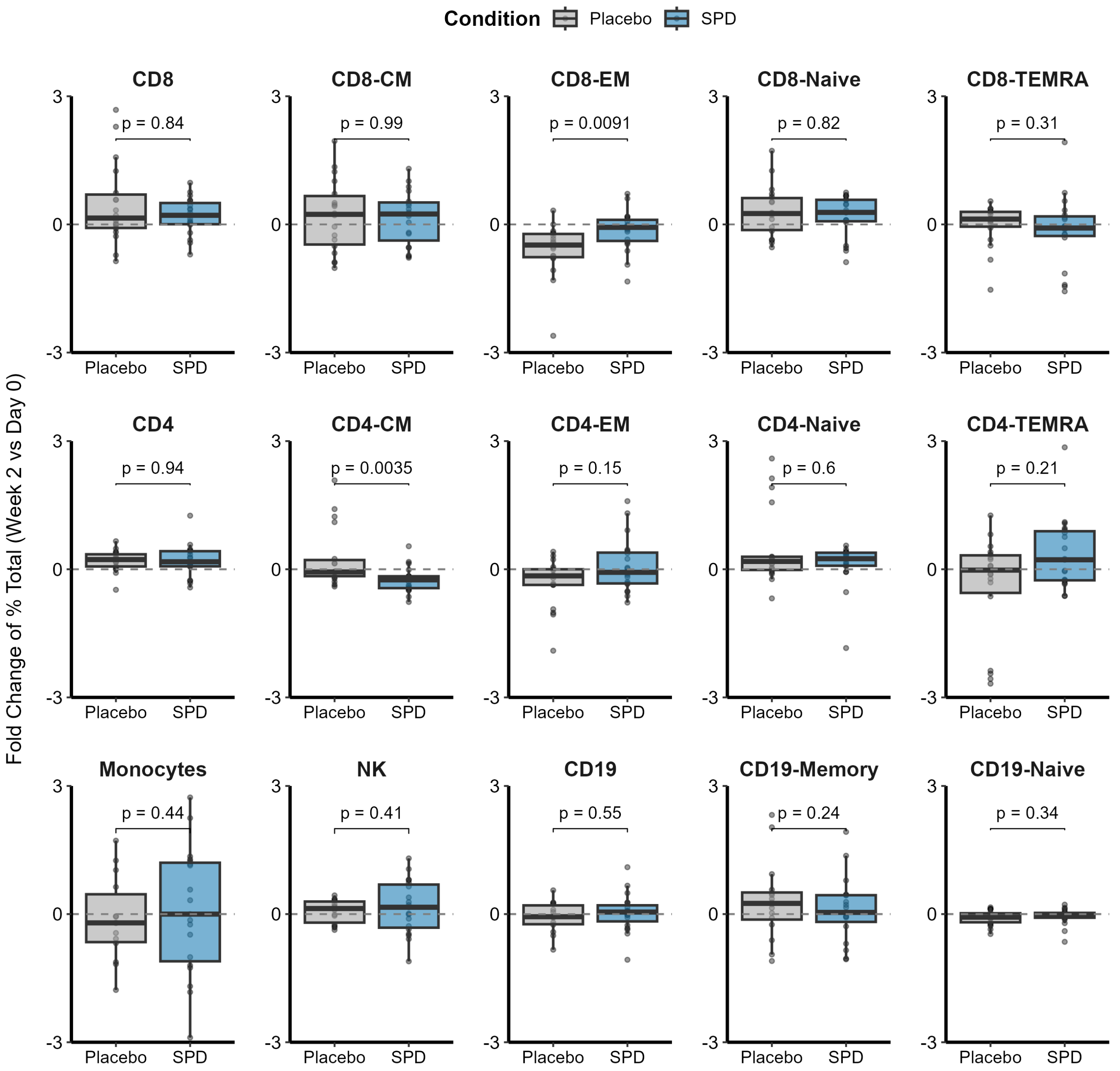

Figure S1: Immune cell subset profiling between Placebo and Spermidine Groups at 2 weeks (log2FC).

Figure S2

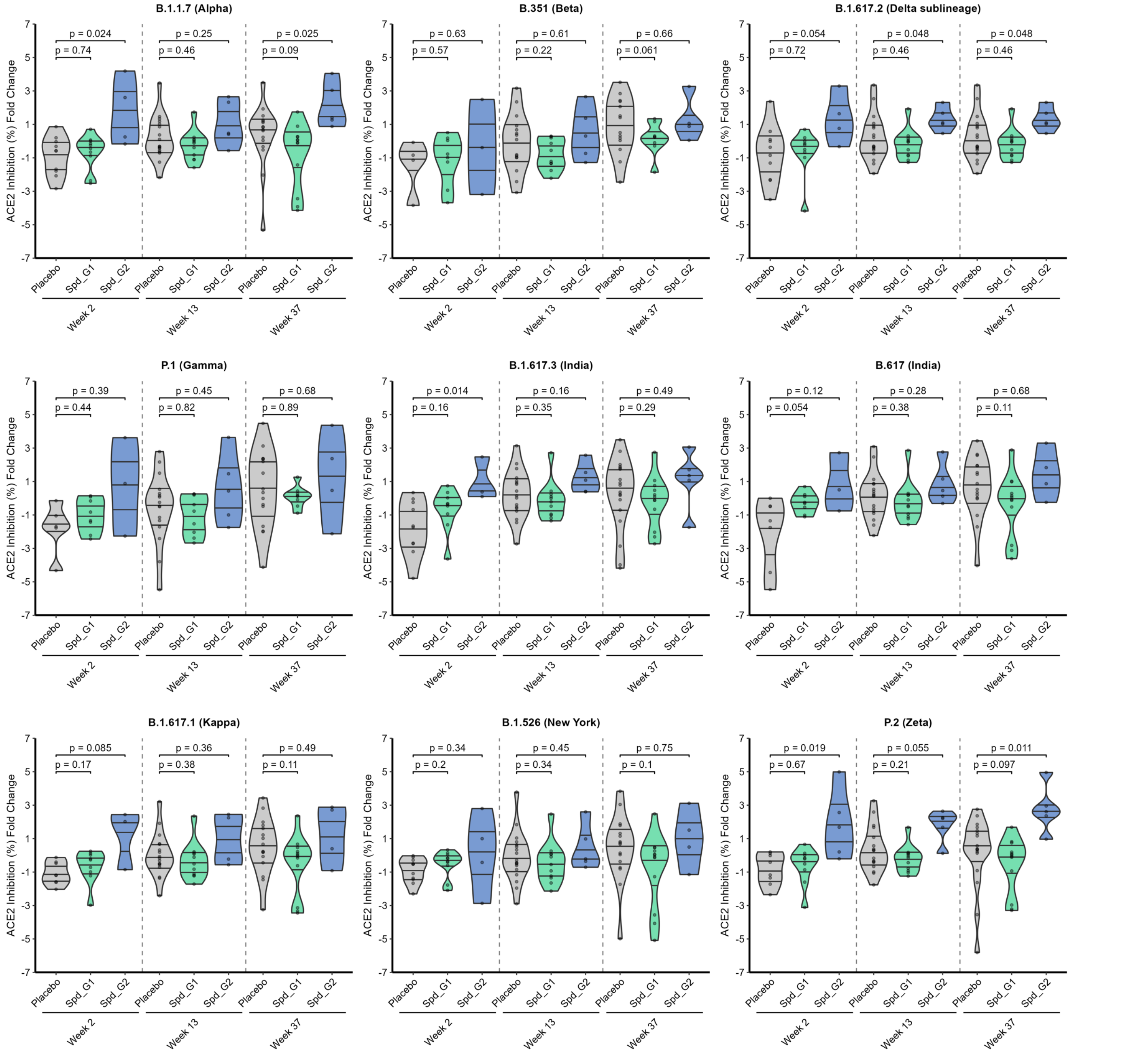

Figure S2: Enhanced ACE2 inhibition responses against SARS-CoV-2 variants following spermidine supplementation.

A-I: ACE2 inhibition (%) log2FC across all groups and timepoints, from neutralisation antibody assay against SARS-CoV-2 variants: **(A)** B.1.617 (India), **(B)** P.2 (Zeta), **(C)** B.1.1.7 (Alpha), **(D)** B.1.351 (Beta), **(E)** B.1.526 (New York), **(F)** B.1.617.2 (Delta), **(G)** B 1.617.1 (Kappa), **(H)** B.1.617.3 (India), **(I)** P.1 (Gamma). Annotations: Week 2 (W2); placebo and spermidine (Spd); group 1 (G1); group 2 (G2). Exact P values are reported. Outliers were retained in all statistical analyses; in some plots, extreme values were omitted from display for clarity. Data are presented as violin plots show median and IQR, with statistical comparisons using the two-sided Wilcoxon rank-sum test. Analyses in R 4.5.0; α = 0.05.

Figure S3.

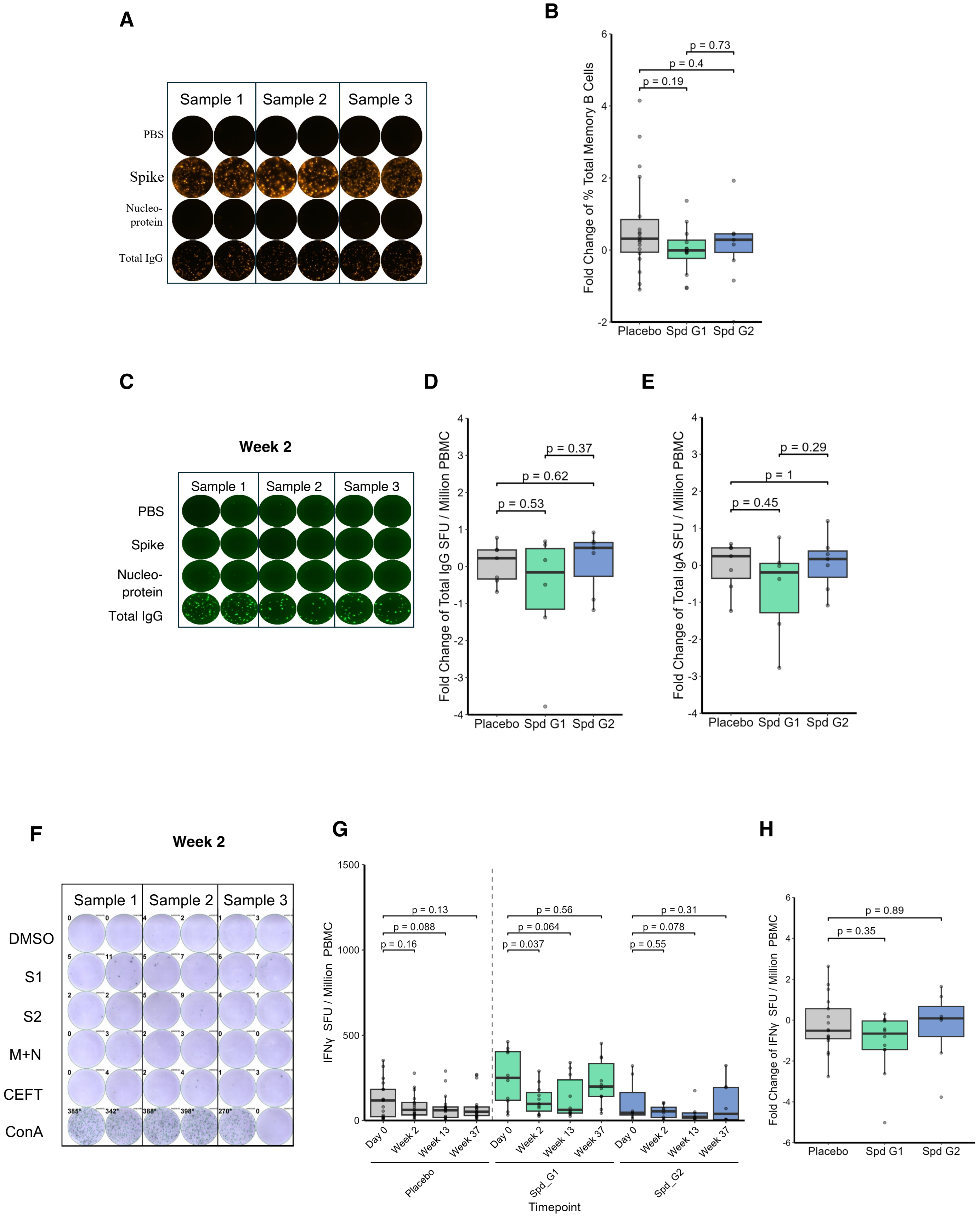
A
Sample 1
Sample 2
Sample 3
PBS
Spike
Nucleo-protein
Total IgG
B
p = 0.73
p = 0.4
p = 0.19
Fold Change of % Total Memory B Cells
Placebo
Spd G1
Spd G2
C
Week 2
Sample 1
Sample 2
Sample 3
PBS
Spike
Nucleo-protein
Total IgG
D
p = 0.37
p = 0.62
p = 0.53
Fold Change of Total IgG SFU / Million PBMC
Placebo
Spd G1
Spd G2
E
p = 0.29
p = 1
p = 0.45
Fold Change of Total IgA SFU / Million PBMC
Placebo
Spd G1
Spd G2
F
Week 2
Sample 1
Sample 2
Sample 3
DMSO
S1
S2
M+N
CEFT
ConA
G
p = 0.13
p = 0.088
p = 0.16
p = 0.56
p = 0.064
p = 0.037
p = 0.31
p = 0.078
p = 0.55
IFNγ SFU / Million PBMC
Day 0
Week 2
Week 13
Week 37
Placebo
Spd_G1
Spd_G2
Timepoint
H
p = 0.89
p = 0.35
Fold Change of IFNγ SFU / Million PBMC
Placebo
Spd G1
Spd G2

Figure S3: Spermidine supplementation does not affect memory B cells, antibody levels, or T cell responses following COVID-19 vaccination.

A: FluoroSpot example image for B cells in spike IgG plates. **B:** Memory B cell ($CD19^{+}$ $CD27^{+}IgD^{low}$) proportion $\log_2$ fold change at 2 weeks. **C:** FluoroSpot image example results for B cells in IgA and IgG plates. **D:** Total IgG (FluoroSpot) $\log_2$ fold change across groups from baseline at 2 weeks. **E:** Total IgA $\log_2$ fold change across groups from baseline at 2 weeks. **F:** ELISpot image example results for T cell plates stimulated with SARS-CoV-2 peptide pools representing the viral S1, S2, membrane (M) and nucleocapsid (N) regions. CEFT peptide pools and concanavalin A (ConA) were used as positive controls. **G:** *Ex vivo* IFN-γ ELISpot response raw data all groups and timepoints to total spike. **H:** *Ex vivo* IFN-γ ELISpot response $\log_2$ fold change at 2 weeks to total spike. Exact P values are reported. Outliers were retained in all statistical analyses; in some plots, extreme values were omitted from display for clarity. Data are presented as Box plots show median, IQR, and 1.5×IQR whiskers, with statistical comparisons using the two-sided Wilcoxon rank-sum test, except for panel F where the Wilcoxon signed-rank test was used. Analyses were conducted in R 4.5.0; $\alpha = 0.05$.

Figure S4

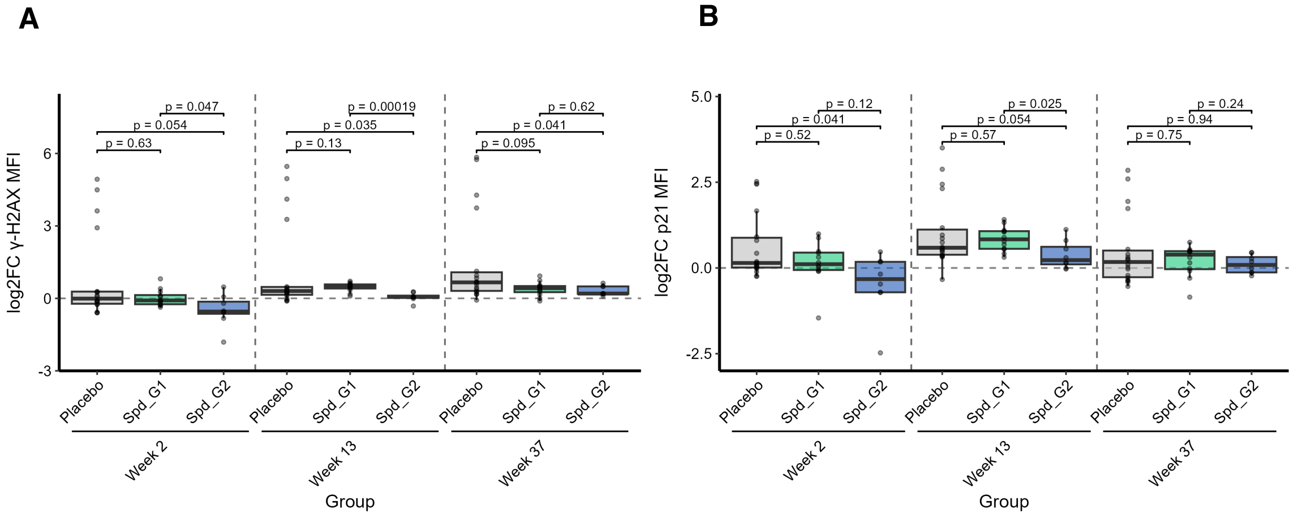

Figure S4: Spermidine treatment promotes senescent cell rejuvenation in vaccine non-responders, (Group 2, 'G2').

A-D: Senescence marker log_2 fold change between day 0/basline and 2, 13 and 37 weeks for **(A)** γH2AX (MFI) **(B)** p-21 (MFI), in placebo (grey), Spd groups responders (G1, green) and non-responders (G2, blue) in PBMCs. For A-B, statistical comparisons were performed using the two-sided Wilcoxon rank-sum test. Statistical significance is indicated by asterisks (*, **, ***, **** for $P < 0.05$, 0.01, 0.001, 0.0001, respectively). Analyses were performed in R 4.5.0 with $\alpha = 0.05$.

Figure S5

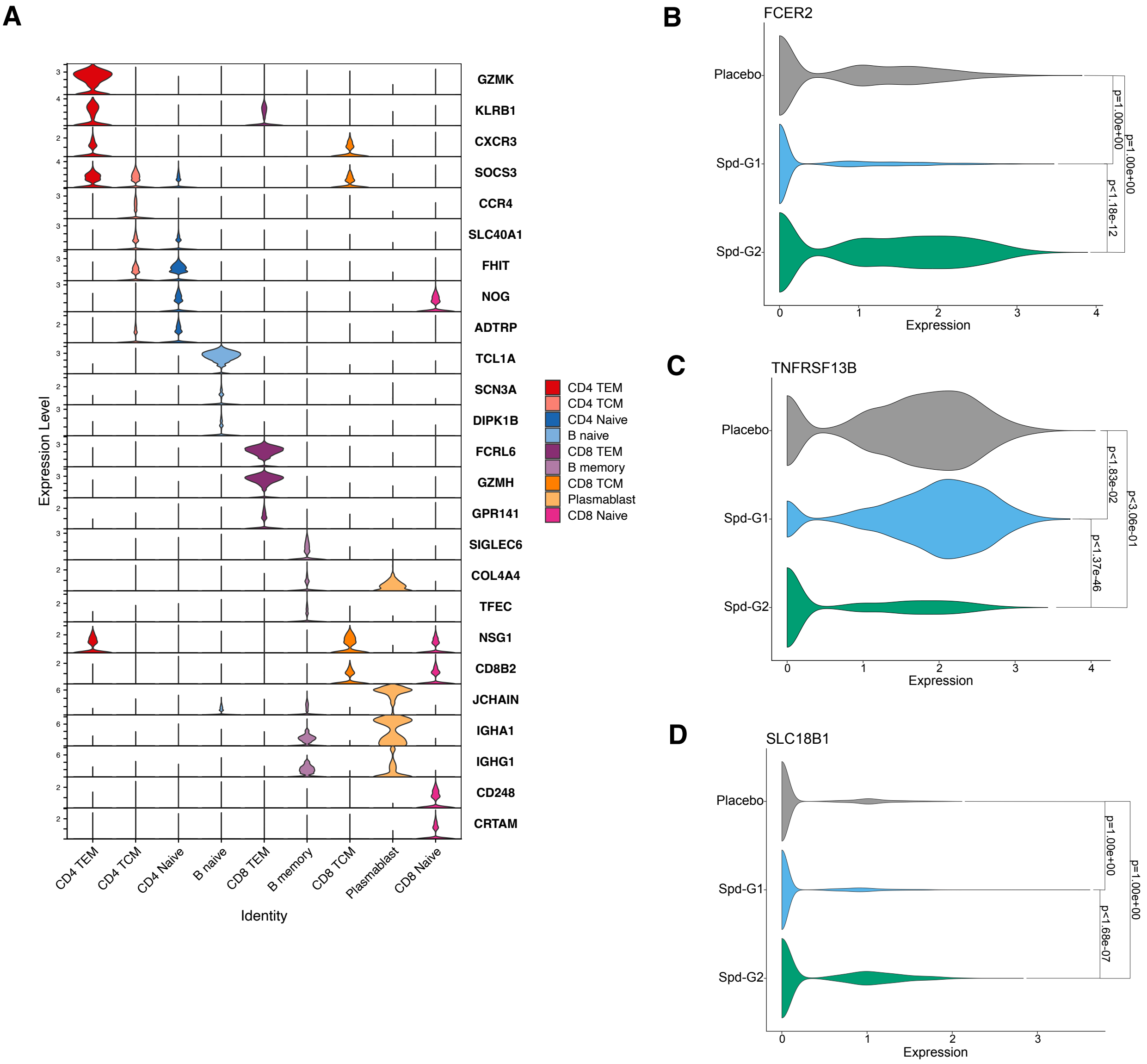

Figure S5: scRNAseq analysis revealed that spermidine treatment induces significant modifications in B cell pathways.

A: Violin plots showing normalized expression of the top three differentially expressed marker genes per identified cell type. **B-D:** Violin plots representing normalised gene expression for select genes differentially expressed between Placebo, G1 and G2 at baseline in the B memory cluster **(B)** FCER2 **(C)** TNFRSF13B **(D)** SLC18B1. Data are presented as violin and dotplots boxplots with statistical comparisons using the Wilcoxon rank-sum tests.

Figure S6

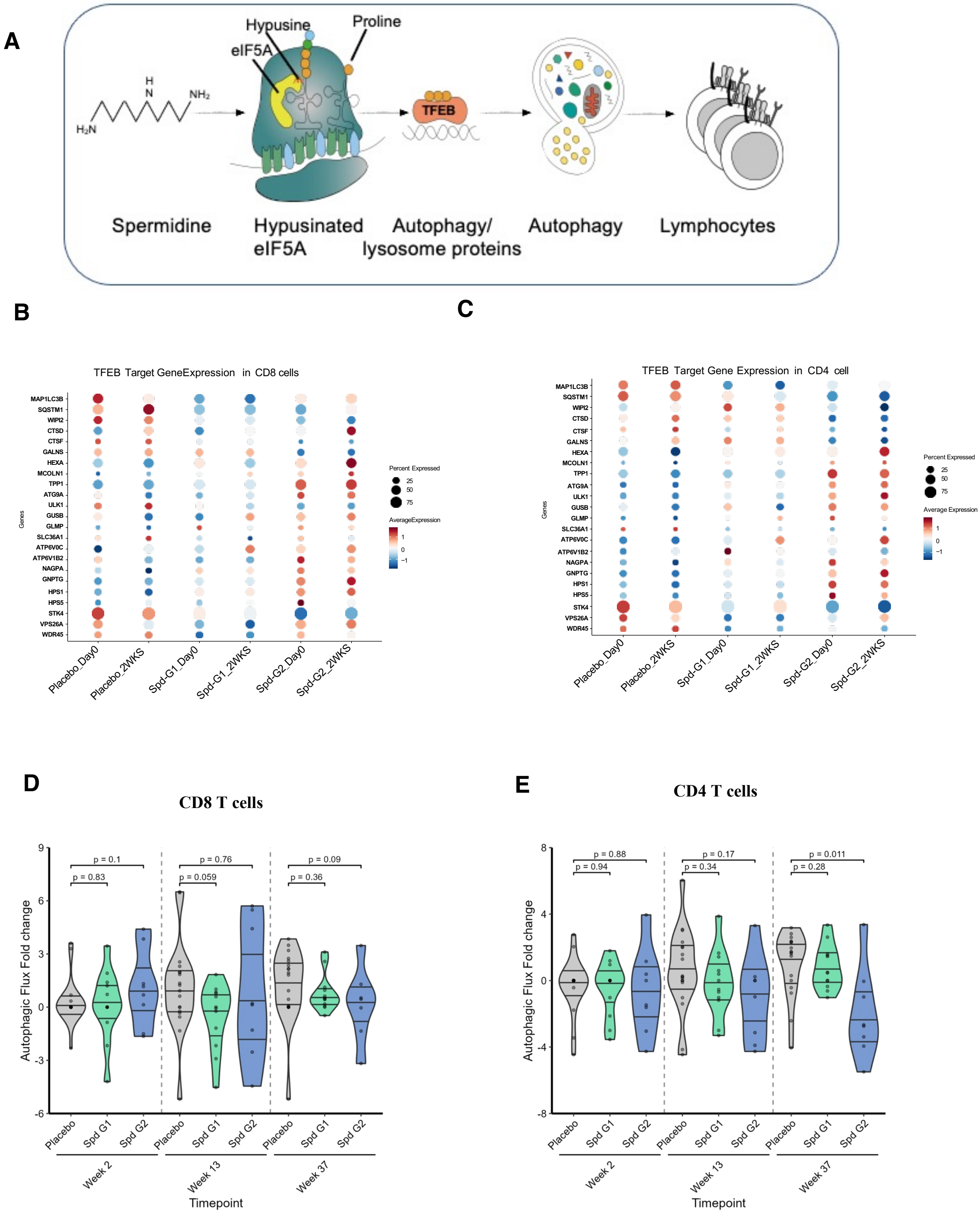

Figure S6: Spermidine supplementation does not induce TFEB or Autophagy in T Cell following COVID-19 vaccination.

A: Schematic representation of the proposed mechanism of action of spermidine supplementation in autophagy activation of immune cells. **B-C:** TFEB target gene expression (scaled) dot heatmaps and pathways of DEGs in **(B)** CD8^{+} T cells **(C)** CD4^{+} T cells. Annotations: Week 2 (W2); placebo and spermidine (Spd); group 1 (G1); group 2 (G2). **D-E:** Autophagic flux $\log_2$ fold change at all timepoints post spermidine supplementation in **(D)** CD8+ T cells and **(E)** CD4^{+} T cells in spermidine Placebo (grey) (Spd) groups 1 (G1, green) and Spd group 2 (G2, blue). Annotations: spermidine (Spd); group 1 (G1); group 2 (G2). Exact P values are reported. Outliers were retained in all statistical analyses; in some plots, extreme values were omitted from display for clarity. Data are presented as violin plots showing median and IQR, with statistical comparisons using the two-sided Wilcoxon rank-sum test. Analyses in R 4.5.0; $\alpha = 0.05$.

Figure S7

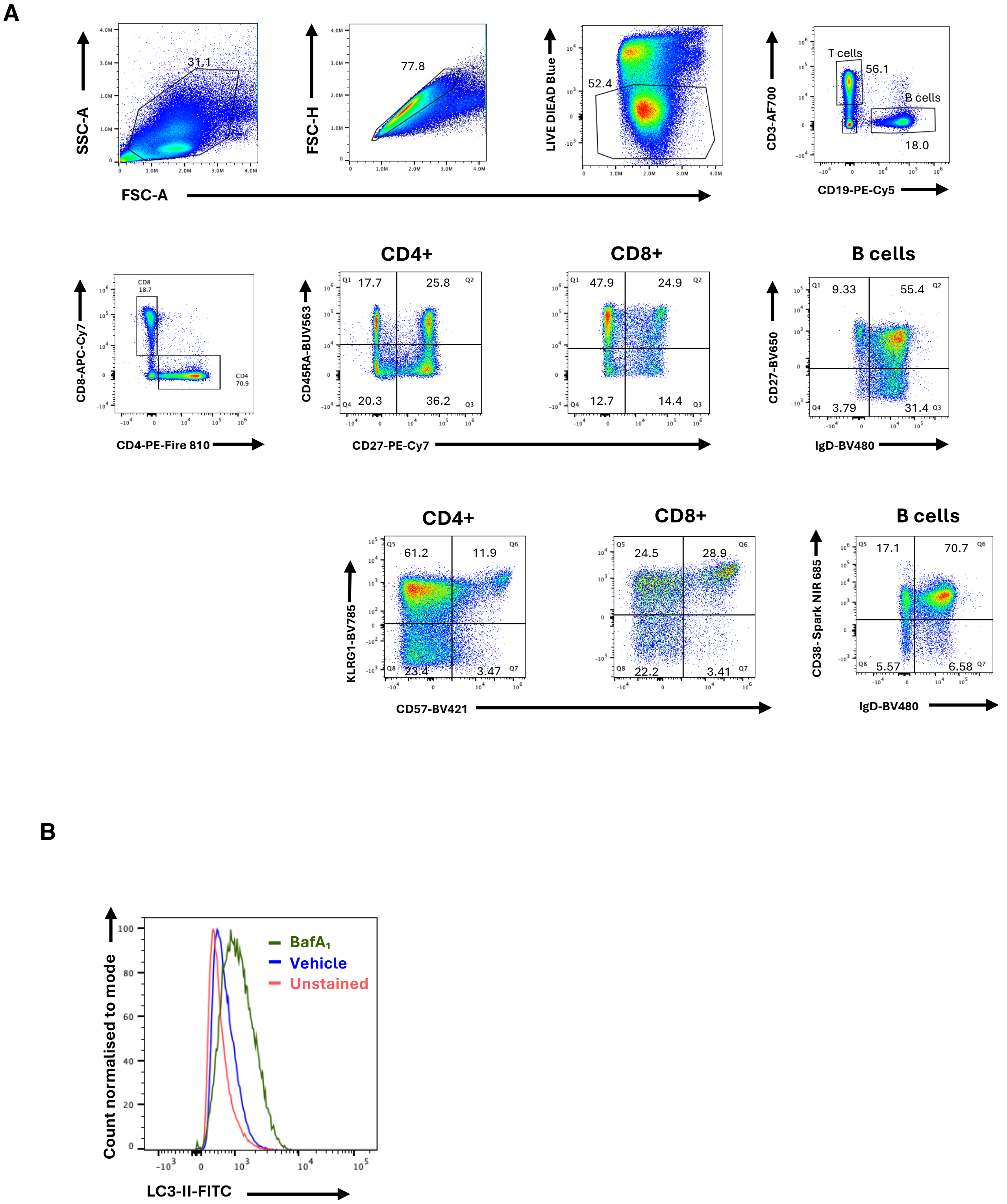

Figure S7: Gating strategies to determine cellular composition of B cells and CD4$^+$ and CD8$^+$ T cells. **A:** Gating strategy for CD3$^+$, CD19$^+$, CD4$^+$, CD8$^+$ subsets within live cells. **B:** Representative flow cytometry-based assay for LC3-II from PBMCs treated with or without bafilomycin A_1 ($BafA_1$) for 2 h prior to staining.

Figure S8

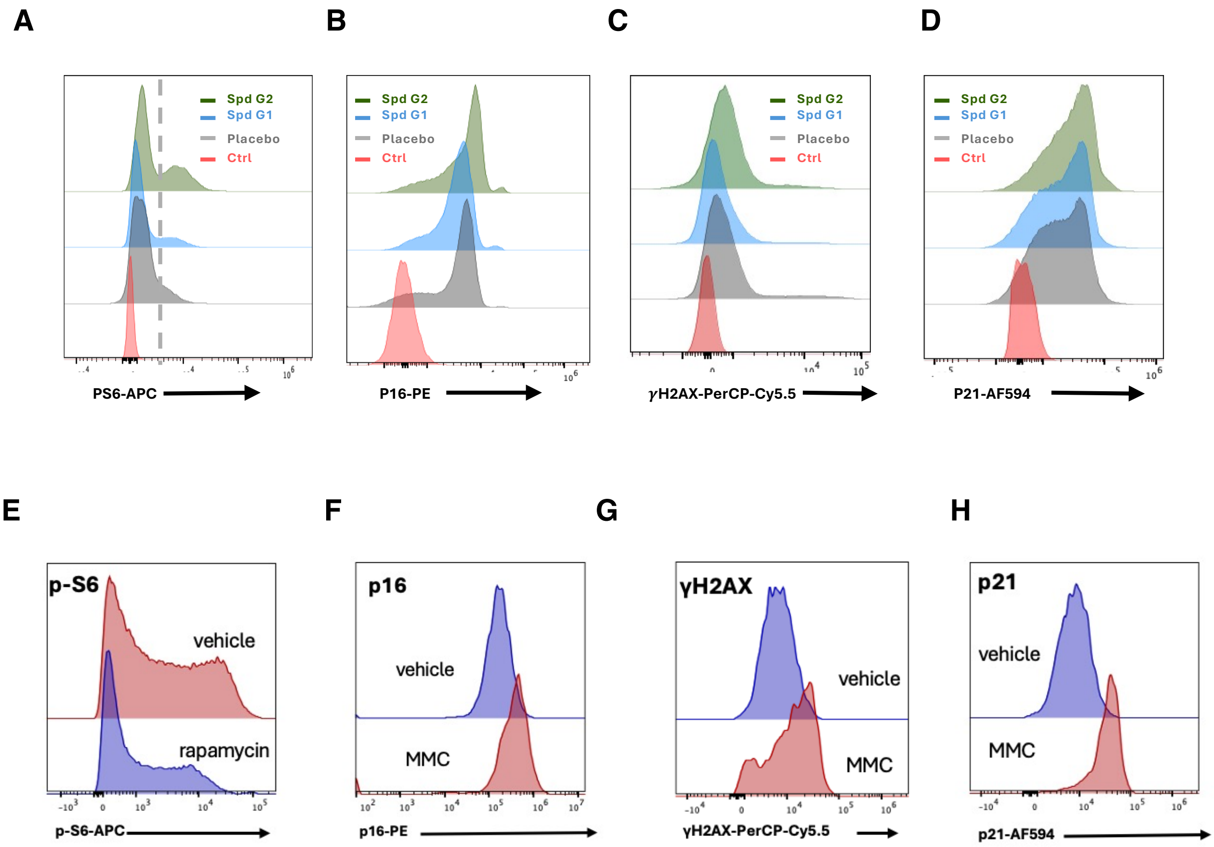

Figure S8: Flow Cytometry analysis of senescence markers at baseline /day0.

A-D: Representative flow cytometry for PBMC stained with antibodies targeting senescence markers, **(A)** pS6 **(B),** p16, **(C)** γ-H2AX and **(D)** p21 in placebo (grey), and the spermidine-treated (Spd) groups split into vaccine responders (G1, green) and non-responders (G2, blue). **E:** mTORC1 activity measured by phospho-S6 (p-S6) levels was assessed in human CD4$^+$ T cells activated for 3 days (1 µg/ml anti-CD3/28) in the presence of vehicle or 10 nM rapamycin (mTORC1 inhibitor). **F-H** Human dermal fibroblasts were treated with 100 nM mitomycin C (MMC) for 6 days and allowed to recover for a further 6 days to induce cellular senescence. Cells were then stained with antibodies targeting senescence markers, **(F)** p16**, (G)** p21, and **(H)** γ-H2AX, and measured by flow cytometry.

Supplementary Table 1: *Adverse reactions and COVID-19 cases.*

	**Placebo (n=18)**	**Spermidine (n=20)**
**Serious adverse reactions (number)**	0	0
**Protocol-related adverse events (number)**	0	0
**PCR confirmed COVID-19 cases adverse events**	4	7
**Timepoint of PCR confirmed COVID-19 cases (number)**		
Day 0/baseline to week 8	0	0
week 9	1	1
week 11	0	1
week 15	1	2
week 19	1	0
week 24	0	2
**Non-COVID19 adverse events**	2	2

Supplementary Table 2A : **Adjusted model (outcome ~ Group + Sex)**
Tests whether sex imbalance confounds the treatment effect.

**Outcome**	**N**	**P_group**	**P_sex**
Anti-spike IgG — Day 0	38	0.1381	0.635
Anti-spike IgG — Week 2	38	0.0488	0.9641
Anti-spike IgG — Week 13	38	0.0553	0.1878
Anti-spike IgG — Week 37	37	0.6115	0.0076
Memory B IgG ELISpot — Day 0	20	0.1654	0.8907
Memory B IgG ELISpot — Week 2	20	0.635	0.8529
nAb % Inhibition (Wuhan) — Day 0	38	0.9157	0.6046
nAb % Inhibition (Wuhan) — Week 2	37	0.0613	0.6732
nAb % Inhibition (Wuhan) — Week 13	35	0.3129	0.2976
nAb % Inhibition (Wuhan) — Week 37	38	0.7671	0.0033*

Supplementary Table 2B : Interaction model (outcome ~ Group × Sex)
Tests whether the treatment effect is modified by sex .

**Outcome**	**N**	**P_group**	**P_sex**	**P_interaction**
Anti-spike IgG — Day 0	38	0.2947	0.804	0.9079
Anti-spike IgG — Week 2	38	0.2936	0.794	0.7455
Anti-spike IgG — Week 13	38	0.2352	0.327	0.931
Anti-spike IgG — Week 37	37	0.38	0.163	0.4656
Memory B IgG ELISpot — Day 0	20	0.3005	0.91	0.8035
Memory B IgG ELISpot — Week 2	20	0.7401	0.949	0.9525
nAb % Inhibition (Wuhan) — Day 0	38	0.9875	0.661	0.9121
nAb % Inhibition (Wuhan) — Week 2	37	0.448	0.887	0.5494
nAb % Inhibition (Wuhan) — Week 13	35	0.3487	0.633	0.7045
nAb % Inhibition (Wuhan) — Week 37	38	0.7793	0.042*	0.9057

Supplementary Table 3: *List of antibodies and reagents.*

Antibody name	Description	Clone	Fluorophore	Source	Code/Catalogue number
CD3	Surface marker for T cells	UCHT1	BUV395	BD Biosciences	563546
CD11c	Surface marker for myeloid cells	B-ly6	BUV496	BD Biosciences	741139
CD45RA	Surface marker for T cells	HI100	BUV563	BD Biosciences	612927
CD19	Surface marker for B cells	SJ25C1	BUV615	BD Biosciences	612990
CD28	Surface marker for T cells	CD28.2	BUV661	BD Biosciences	741635
HLA-DR	Surface marker for monocytes	L203.rMAb	BUV737	BD Biosciences	752496
CD57	Surface marker for T cells and NK cells	NK-1	BV421	BD Biosciences	563896
NKG2A	Surface marker for NK cells	S19004C	Pacific Blue	BioLegend	375110
IgD	Surface marker for B cells	IA6-2	BV480	BD Biosciences	566187
CD45RO	Surface marker for T cells	UCHL1	BV510	BioLegend	304245
CD56	Surface marker for NK cells	HCD56	BV570	BioLegend	318329
CD27	Surface marker for T cells and B cells	O323	BV650	BioLegend	302827
KLRG1	Surface marker for T cells	2F1/KLRG1	BV785	BioLegend	138429
CCR7	Surface marker for T cells	G043H7	PerCP-Cy5.5	BioLegend	353219
CD16	Surface marker for monocytes	3G8	PE-Dazzle594	BioLegend	302053
CD127	Surface marker for T cells	A019D5	PE-Fire 700	BioLegend	351365
CD4	Surface marker for T cells	SK3	PE-Fire 810	BioLegend	344677

CD8	Surface marker for T cells	HIT8a	APC-Cy7	BioLegend	300925
CD14	Surface marker for B cells and monocytes	63D3	APC-Fire 810	BioLegend	367155
Perforin	Intracellular marker	B-D48	PE-Cy7	BioLegend	353316
Granzyme B	Intracellular marker	GB11	PE	eBioscience	12-8899-41
IFNγ	Intracellular marker	4S.B3	Ef506	ThermoFisher	69-7319-42
TNFα	Intracellular marker	MAb11	FITC	BioLegend	502906
IL-2	Intracellular marker	MQ1-17H12	BV650	BioLegend	500333
Phospho-S-6	Intracellular marker	cupk43k	APC	ThermoFisher Scientific	417-9007-42
P21	Intracellular marker	12D1	Alexa Fluor® 594	Cell Signaling	11850
P16	Intracellular marker	EPR1473	PE	abcam	ab209579
γH2Ax	Intracellular marker	N1-431	PerCP-Cy™5.5	BD Biosciences	564718
